# LaminA/C regulates epigenetic and chromatin architecture changes upon aging of hematopoietic stem cells

**DOI:** 10.1186/s13059-018-1557-3

**Published:** 2018-11-07

**Authors:** Ani Grigoryan, Novella Guidi, Katharina Senger, Thomas Liehr, Karin Soller, Gina Marka, Angelika Vollmer, Yolanda Markaki, Heinrich Leonhardt, Christian Buske, Daniel B. Lipka, Christoph Plass, Yi Zheng, Medhanie A. Mulaw, Hartmut Geiger, Maria Carolina Florian

**Affiliations:** 1 Institute of Molecular Medicine and Stem Cell Aging, University of Ulm, Albert-Einstein-Allee 11c, 89081 Ulm, Germany; 2 Department of Biology II and Center for Integrated Protein Science Munich (CIPSM), Ludwig Maximilians University Munich, Großhaderner Strasse 2, 82152 Planegg-Martinsried, Germany; 3 Institute of Experimental Cancer Research, Comprehensive Cancer Center Ulm, University Hospital Ulm, Albert-Einstein-Allee 11, 89081 Ulm, Germany; 4 Institute of Human Genetics, Jena University Hospital, Friedrich Schiller University, Kollegiengasse 10, 07743 Jena, Germany; 5 Regulation of Cellular Differentiation Group, INF280, 69120 Heidelberg, Germany; 6 Division of Epigenomics and Cancer Risk Factors, German Cancer Research Center (DKFZ), INF280, 69120 Heidelberg, Germany; 7 Division of Experimental Hematology and Cancer Biology, Cincinnati Children’s Hospital Medical Center and University of Cincinnati, Cincinnati, OH USA; 8 Center of Regenerative Medicine in Barcelona (CMRB), Hospital Duran i Reynals, Gran Via de l’Hospitalet, 199-203, L’Hospitalet de Llobregat, 08908 Barcelona, Spain

**Keywords:** Hematopoietic stem cell (HSC), Aging, Chromatin architecture, LaminA/C, Chromosome 11

## Abstract

**Background:**

The decline of hematopoietic stem cell (HSC) function upon aging contributes to aging-associated immune remodeling and leukemia pathogenesis. Aged HSCs show changes to their epigenome, such as alterations in DNA methylation and histone methylation and acetylation landscapes. We previously showed a correlation between high Cdc42 activity in aged HSCs and the loss of intranuclear epigenetic polarity, or epipolarity, as indicated by the specific distribution of H4K16ac.

**Results:**

Here, we show that not all histone modifications display a polar localization and that a reduction in H4K16ac amount and loss of epipolarity are specific to aged HSCs. Increasing the levels of H4K16ac is not sufficient to restore polarity in aged HSCs and the restoration of HSC function. The changes in H4K16ac upon aging and rejuvenation of HSCs are correlated with a change in chromosome 11 architecture and alterations in nuclear volume and shape. Surprisingly, by taking advantage of knockout mouse models, we demonstrate that increased Cdc42 activity levels correlate with the repression of the nuclear envelope protein LaminA/C, which controls chromosome 11 distribution, H4K16ac polarity, and nuclear volume and shape in aged HSCs.

**Conclusions:**

Collectively, our data show that chromatin architecture changes in aged stem cells are reversible by decreasing the levels of Cdc42 activity, revealing an unanticipated way to pharmacologically target LaminA/C expression and revert alterations of the epigenetic architecture in aged HSCs.

**Electronic supplementary material:**

The online version of this article (10.1186/s13059-018-1557-3) contains supplementary material, which is available to authorized users.

## Background

Somatic stem cell activity is critical for tissue regeneration, and the decline in stem cell function upon aging contributes to tissue attrition [1]. In particular, hematopoiesis in the elderly presents with an aging-associated immune remodeling that manifests as a decrease in red blood cells (RBC) and lymphoid cells (T and B cells) in bone marrow (BM) and peripheral blood (PB) as well as an increase in myeloid cells and in the incidence of leukemia. These aging-associated changes are, at least in part, a consequence of intrinsic aging of hematopoietic stem cells (HSCs) [1–7].

Upon aging, the HSC epigenome undergoes an “epigenetic drift” [8–10] that comprises alterations in the global and local DNA/histone methylation and histone acetylation landscape [9, 11–15]. The meaning and the consequences of this epigenetic drift for HSC aging are overall controversially discussed. For example, it was reported that there is an increase of domains that are marked by both active (H3K4me3) and repressive (H3K27me3) histone modifications in aged compared to young HSCs [14]. In addition, specific alterations of DNA methylation were also shown to occur upon HSC aging at genomic regions targeting genes not expressed in stem cells but only in downstream progenitor and effector cells [9, 15]. These observations might imply that the contribution of epigenetic changes to HSC aging could be to some extent beyond the immediate direct regulation of gene transcription in stem cells. Furthermore, a mechanism explaining the general impact of aging on any epigenetic alterations is still largely missing, preventing the development of a targeted approach to eventually improve aged stem cell function.

**Fig. 1:**
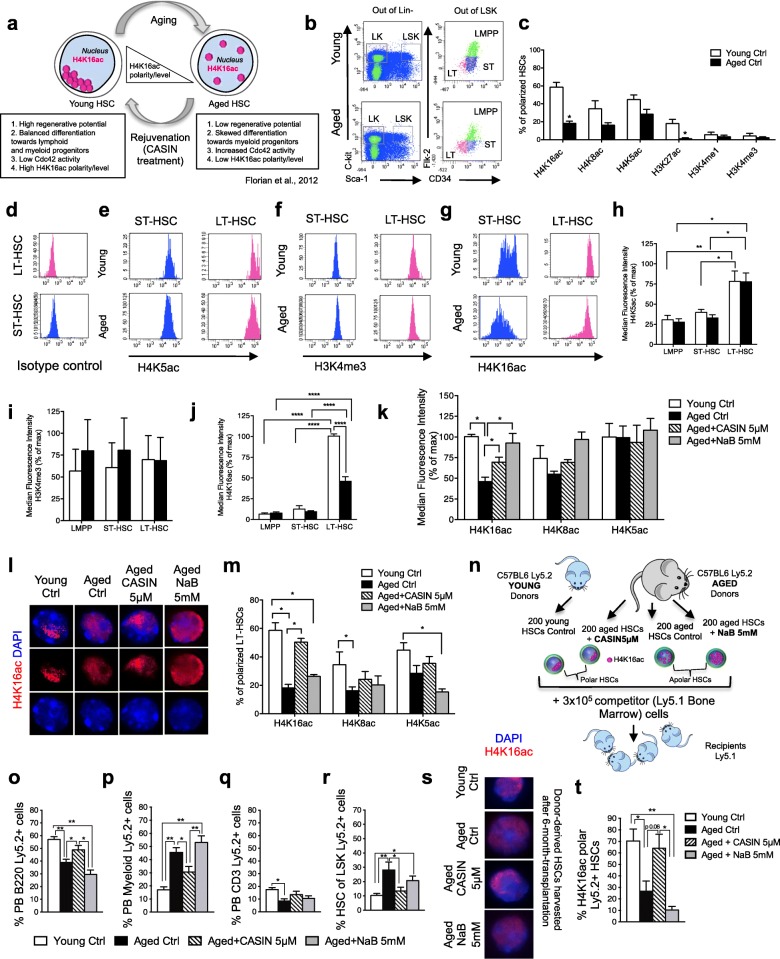
The change in the spatial distribution of H4K16ac upon aging is linked to a change in HSC function. **a** Aging of HSCs. Cartoon scheme of the main previous findings [16]. **b** Representative FACS dot plots of HSCs (Lin-c-kit+Sca-1+Flk2-CD34-), ST-HSCs (Lin-c-kit+Sca-1+Flk2-CD34+), LMPPs (Lin-c-kit+Sca-1+Flk2+CD34+), and LSKs (Lin-c-kit+Sca-1+) gating strategy of young and aged lineage-depleted BM cells. **c** Percentage of young and aged HSCs with a polar distribution of H4K16ac, H4K8ac, H4K5ac, H3K27ac, H3K4me1, and H3K4me3; *n* = 3–4 biological repeats; ~ 150–200 single HSCs scored per sample in total; *p < 0.001. **d** Representative FACS histograms of an isotype control stained sample. Gates for LT-HSCs and ST-HSCs. Representative FACS histograms of H3K5ac (**e**), H3K4me3 (**f**), and H4K16ac (**g**) of young and aged samples. LT-HSCs and ST-HSCs gates are shown. Median fluorescence intensity of H3K5ac (**h**), H3K4me3 (**i**), and H4K16ac (**j**) plotted as a percentage of young control in young and aged HSCs, ST-HSCs, and LMPPs. Shown are mean values + 1 SE; *n* = 4–7 biological repeats; *p < 0.05. **k** Median fluorescence intensity of H4K16ac, H4K8ac, and H4K5ac plotted as a percentage of young control in young, aged, aged CASIN-treated (5 μM, 16 h), and aged NaB-treated (5 mM, 16 h) HSCs. Shown are mean values + 1 SE; *n* = 4 biological repeats; *p < 0.05. **l** Representative distribution of H4K16ac in young, aged, aged CASIN-treated (5 μM, 16 h), and aged NaB-treated (5 mM, 16 h) HSCs. Bar = 2 μm. Panels show DAPI (nucleus, blue), H4K16ac (red). **m** Percentage of young, aged, aged CASIN-treated (5 μM, 16 h), and aged NaB-treated (5 mM, 16 h) HSCs with a polar distribution of H4K16ac; *n* = 3–4 biological repeats; ~ 150–200 single HSCs scored per sample in total; *p < 0.05. **n** Schematic representation of the experimental setup for HSC transplantation. Percentage of B220^+^ (**o**), myeloid (Gr1^+^, Mac1^+^, and Gr1^+^Mac1^+^) (**p**), and CD3^+^ (**q**) cells among Ly5.2^+^ donor-derived cells in peripheral blood (PB) of recipient Ly5.1^+^ mice; *p < 0.05, **p < 0.01; *n* = 12–19. **r** Percentage of HSCs among donor-derived Ly5.2^+^ LSKs in BM of recipient Ly5.1^+^ mice; *p < 0.05, **p < 0.01; *n* = 12–19. **s** Representative distribution of H4K16ac in donor-derived Ly5.2^+^ HSCs sorted from mice after 24 weeks from transplant with young, aged, aged CASIN-treated (5 μM, 16 h), and aged NaB-treated (5 mM, 16 h) HSCs. Bar = 2 μm. Panels show DAPI (nucleus, blue), H4K16ac (red). **t** Percentage of H4K16ac-polarized donor-derived Ly5.2^+^ HSCs sorted from mice after 24 weeks from transplant with young, aged, aged+CASIN 5 μM, and aged+NaB 5 mM HSCs; *n* = 3 biological repeats; ~ 100–150 single HSCs scored per sample in total; *p < 0.05, **p < 0.01

Previously, we reported that young and aged HSCs differ in both the level and the polar distribution (epigenetic polarity or “epipolarity”) of histone 4 acetylated on lysine 16 (H4K16ac). H4K16ac levels and epipolarity in HSCs are regulated by the activity of the small RhoGTPase Cdc42. Indeed, a reduction of the aging-associated elevated Cdc42 activity by a small molecule compound CASIN (*C*dc42-*a*ctivity *s*pecific *in*hibitor) restores H4K16ac levels and epipolarity and rejuvenates the function of chronologically aged HSCs (Fig. 1a) [1, 16, 17]. These data imply a tight connection between a change in levels and epipolarity of H4K16ac and aging and rejuvenation of HSCs (Fig. 1a). The 3D distribution of specific histone post-translational modifications (PTMs) correlates with the organization of chromatin and the architecture of the nucleus [18–20]. Therefore, we hypothesize that alterations in H4K16ac in aged HSCs might be linked not only to a possible regulation of gene transcription, but also to the organization of chromatin and/or chromosome architecture. So far, it remains elusive whether chromatin and/or chromosome architecture is altered upon HSC aging, which mechanisms underlie these epigenetic alterations, and eventually whether they might represent a target in aged stem cells.

Here, we report that changes in polarity of H4K16ac are tightly linked to the alteration in the localization of specifically chromosome 11 within the nucleus of HSCs upon aging, while alterations in the level of H4K16ac do not generally correlate with epipolarity nor with the function of HSCs. Mechanistically, we demonstrate that the nuclear envelope protein LaminA/C causatively confers changes in the nuclear volume and shape, in H4K16ac epipolarity, and in the localization of chromosome 11 homologs upon HSC aging in response to changes in the activity levels of Cdc42. Consistently, HSCs deficient of LaminA/C present with premature aging-like phenotypes that extend to alterations in nuclear volume and shape, chromosome 11 homologs localization, and H4K16ac epipolarity. Collectively, our data unravel a novel Cdc42-LaminA/C axis that is implicated in preserving chromatin architecture and function of hematopoietic stem cells upon aging.

## Results and discussion

### The change in the spatial distribution of H4K16ac upon aging is linked to a change in HSC function

Aged HSCs show both a change in the overall level of H4K16ac and a change in the spatial distribution of H4K16ac within the nucleus (Additional file 1: Figure S1a), which we described as a loss of epigenetic polarity (or epipolarity) [16] (Fig. 1a). Therefore, we also asked whether in general other histone PTMs localize differently in HSCs upon aging (the representative gating strategy is depicted in Fig. 1b) or not and whether the level of a histone PTM and its spatial distribution within the nucleus are linked.

H4K8ac, H4K5ac, and H3K27ac also displayed a polar distribution in young HSCs, but to an overall lower frequency compared to H4K16ac, and only H3K27ac showed a significant further decline in the frequency of cells with a polar distribution upon aging (17.9 ± 4.6% in young HSCs and 1.1 ± 1.1% in aged). H3K4me1 and H3K4me3 displayed no polar distribution in either young or aged HSCs (Fig. 1c and Additional file 1: Figure S1b-c). The data imply that the reduction of epipolarity in HSCs upon aging is selective and predominantly affects H4K16ac. The high frequency of H4K16ac epipolarity in young HSCs is also specific to the more primitive stem cell population, since the level of H4K16ac epipolarity in slightly more differentiated short-term HSCs (ST-HSCs, sorted as lineage- c-kit+ sca1+ CD34+ Flk2- BM cells; gating in Fig. 1b) was very low and not altered upon aging (Additional file 1: Figure S1d).

Next, we asked whether the levels of PTMs change in hematopoietic stem and progenitor cells (LT-HSCs, ST-HSCs, and lymphoid multipotent progenitors or lymphoid-primed multipotent progenitors (LMPPs), gated as lineage- c-kit+ sca1+ CD34+ Flk2+ BM cells; see Fig. 1b for the gating strategy) upon aging. By intracellular flow cytometry, three distinct patterns were observed. In the first one (that applied to H4K5ac), the level of histone PMT was distinctly high in stem cells and was not altered upon aging (Fig. 1d, e, h). The second pattern (comprising H3K4me1, H3K4me3, H4K8ac, and H3K27ac) displayed no stem cell- and aging-specific changes in the level (Fig. 1f, i and Additional file 1: Figure S1e-j). The third pattern was defined by H4K16ac, for which the level was distinctly high in stem cells and decreased with age in an HSC-specific way (Fig. 1g, j). Among the PTMs analyzed, H4K16ac is thus distinct with respect to both changes in polarity and level in HSCs upon aging. In addition, our data imply that in general, changes in the distribution and amount of histone PTMs upon aging do not affect all PTMs uniformly.

The data do not reveal whether the change in the level of H4K16ac or the change in its distribution or a combination of both correlates with changes in the function of HSCs upon aging. To experimentally address this question, we increased the level of H4K16ac in aged HSCs to the level seen in young HSCs via in vitro treatment of aged HSCs with 5 mM sodium butyrate (NaB) (Fig. 1k and Additional file 1: Figure S1k). NaB is a broad inhibitor of histone deacetylases (HDACs) [21–23]. The level of H4K5ac and H4K8ac in aged HSCs was not significantly affected by NaB treatment. Therefore, the effect of NaB treatment in HSCs was relatively specific to H4K16ac. Interestingly, as also previously reported [16], inhibition of Cdc42 activity via CASIN at a concentration that resulted in the rejuvenation of aged HSC function increased the level of H4K16ac in stem cells as well, but not to the level achieved by NaB treatment (Fig. 1k). While the treatment with CASIN restored H4K16ac epipolarity, treatment with NaB did not affect epipolarity in aged HSCs (Fig. 1l, m), and aged NaB-treated HSCs remained apolar for H4K16ac. Therefore, the level of H4K16ac does not generally correlate with its distribution in HSCs, and both parameters (level and polarity) are likely independent of each other.

Next, we investigated whether increasing the level of H4K16ac in aged HSCs without changing H4K16ac epipolarity via NaB treatment improves the function of aged HSCs. To this end, 200 aged NaB-treated Ly5.2^+^ HSCs were competitively transplanted alongside 3 × 10^5^ Ly5.1^+^ bone marrow (BM)-supporting cells into lethally irradiated Ly5.1^+^ recipient mice (Fig. 1n). As a control, 200 young, aged, and aged CASIN-treated Ly5.2^+^ HSCs were competitively transplanted into additional sets of lethally irradiated Ly5.1^+^ recipient mice. Twenty-four-week post-transplant animals that received aged HSCs presented with the anticipated aging-associated shift towards more myeloid cells in PB and BM, an increase in the frequency of donor-derived HSCs, and an overall lower donor cell engraftment compared to young HSCs (Fig. 1o–r and Additional file 1: Figure S1l-s) [5, 16, 24, 25]. The contribution to the B- and myeloid cell pool (Fig. 1o, p), stem cell frequency (Fig. 1r), and polarity for H4K16ac in HSCs (Fig. 1s, t) of donor-derived cells in mice transplanted with young and aged CASIN-treated HSCs was almost indistinguishable from each other and significantly different from aged HSCs (also Additional file 1: Figure S1l-**s**). CASIN-treated aged HSCs were thus rejuvenated (as previously reported [16] and here independently reproduced). To note, the re-distribution (repolarization) of H4K16ac in aged HSCs upon CASIN treatment ex vivo was long-term stably transmitted to daughter stem cells, since HSCs harvested from recipients 6 months after transplantation retained a high frequency of H4K16ac epipolarity (Fig. 1s, t).

In contrast, HSCs treated with NaB, which restored the level of H4K16ac in aged HSCs to the level reported for young HSCs (Fig. 1k), without altering its polar distribution (Fig. 1l, m), remained functionally indistinguishable from aged HSCs for all parameters measured (Fig. 1o–t) and were thus not rejuvenated. Therefore, the distribution but not the amount of H4K16ac strongly correlates with aging and rejuvenation of HSCs.

### Changes in H4K16ac epipolarity are linked to changes in chromosome 11 homologs distribution

**Fig. 2:**
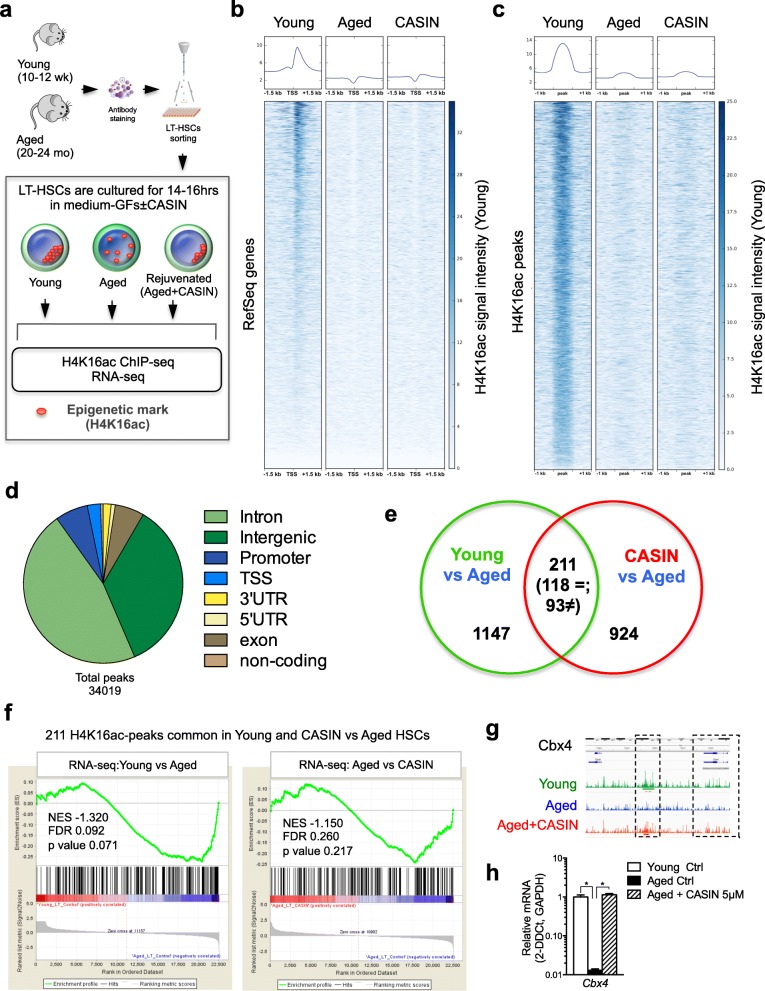
Changes in H4K16ac epipolarity are linked to changes in chromosome 11 homolog distribution. **a** Schematic representation of the experimental setup. Heatmaps and profiles show input-corrected H4K16ac signals ± 1.5 kb from TSS (**b**) and at H4K16ac peaks ± 1.0 kb (**c**). TSS and peak regions were ranked by the signal intensity in young HSCs. Summary profile of *n* = 3 biological repeats. Each sample was generated by pooling 50,000 sorted HSCs that were incubated 16 h in HBSS+ 10% FBS+P/S±CASIN 5 μM at 3% O_2_. **d** Pie chart showing genomic annotations of the total 34,019 H4K16ac peaks in young, aged HSCs, and aged+CASIN 5 μM HSCs. ChIP-seq data are available at GEO Series accession number GSE120232. **e** Venn diagram showing results of the cross-analysis of differentially bound H4K16ac peaks between young and aged HSCs (1358 peaks), aged+CASIN 5 μM, and aged HSCs (1135 peaks). Two hundred eleven differentially bound peaks were overlapping, and of these, 118 showed the same direction of change. **f** Gene Set Enrichment Analysis (GSEA) for the 211 differentially bound H4K16ac peaks overlapping between young and CASIN HSCs as compared to aged HSCs in both young vs aged and CASIN vs aged RNA-seq dataset. RNA-seq data are available at GEO Series accession number GSE119466. **g**–**h** UCSC browser track showing H4K16ac signal at the *Cbx4* locus and real-time PCR transcript level for *Cbx4* in young, aged, and aged+CASIN 5 μM HSCs: *n* = 3 biological repeats, *p < 0.0001

To further investigate the duality between changes in levels and spatial distribution of H4K16ac, we performed chromatin immunoprecipitation sequencing (ChIP-seq) in young, aged, and aged CASIN-treated (rejuvenated) HSCs alongside RNA-seq profiling on the same sample set, to inform on transcriptome changes possibly associated to H4K16ac (Fig. 2a). In general, the H4K16ac ChIP-seq profile in HSCs was characterized by very broad and flat regions of enrichment (see as an example Additional file 1: Figure S2a). Thirty-four thousand nineteen unique H4K16ac-associated peaks were identified from all the samples. Overall, signal intensity was decreased in aged HSCs compared to young, consistent with a reduced level of H4K16ac upon aging, and only very slightly restored in aged CASIN-treated HSCs both at TSS and at all the other genomic regions (Fig. 2b, c). Most of the peaks were associated with intron and intergenic regions, and only a minority was found at TSS and promoters (2.6% and 6.6%, respectively, Fig. 2d). We identified 1358 H4K16ac peaks differentially enriched in young vs aged HSCs and 1135 different peaks when comparing CASIN vs aged samples (Additional file 1: Figure S2b), and their overlap resulted in 211 peaks (Fig. 2e). Out of these, 118 showed the same direction of change (gain) for both young and aged CASIN-treated compared to aged HSCs (see as an example the browser tracks for *Pten* and *Actb*; Additional file 2: Table S1 and Additional file 1: Figure S2c). Using RNA-seq profiling data (Additional file 3: Table S2), we performed principal component analysis (PCA) based between-group analysis (bga) and observed that aged CASIN-treated HSC samples lie equidistantly between young and aged samples (Additional file 1: Figure S2d and Additional file 4: Table S3). The distance between young and aged CASIN-treated samples reduced when we selected differentially expressed genes with increasing degree of stringency (*p* value < 0.05; no false discovery rate (no FDR) adjustment, FDR with Benjamini-Hochberg, and FDR with Bonferroni adjustment), indicating that genes that are differentially expressed between young and aged HSCs are highly similar to those in the aged CASIN-treated vs aged HSC comparison (Additional file 1: Figure S2d and Additional file 4: Table S3). Similarly, heatmap based on unsupervised hierarchical clustering further showed that aged CASIN-treated HSCs clustered closer to young HSCs than to aged HSCs (Additional file 1: Figure S2e). The number of differentially expressed genes when comparing young and aged HSCs was 530 while 387 genes were differentially regulated in the aged CASIN-treated HSCs and aged HSC comparison (Additional file 4: Table S3; list of differentially expressed genes after FDR adjustment are also provided). Gene ontology analyses did not reveal enrichment for the hematopoietic system- or hematopoietic stem cell-related signature (data not shown). When overall RNA-seq data were correlated to overall H4K16ac ChIP-seq-associated genes, correlation plots displayed no association between overall gene expression and global H4K16ac mark deposition (Additional file 1: Figure S2f-g). Gene Set Enrichment Analysis (GSEA) by taking the 211 ChIP-seq peaks overlapping between young and CASIN as compared to aged (Fig. 2e) showed both in young vs aged and CASIN vs aged RNA-seq dataset, no significant enrichment between gene expression and H4K16ac (normalized enrichment score, NES, − 1.33 and − 1.15, respectively) (Fig. 2f). A similar conclusion was obtained when considering the 1358 young vs aged and the 1135 CASIN vs aged H4K16ac-associated peaks (Additional file 1: Figure S2b and h-i). Therefore, there is no correlation of gene expression (both for overall expression and differentially expressed genes) and H4K16ac mark deposition changes in young and aged CASIN-treated HSCs compared to aged HSCs.

However, among the 211 H4K16ac-associated peaks common to young and CASIN samples compared to aged HSCs (Fig. 2e and Additional file 2: Table S1), we identified some selected genes known to be relevant for HSCs and further tested their expression levels by real-time PCR, for example, *Cbx4*, which has been linked to HSC differentiation potential, *Hoxb4*, which is known to play an important role in HSC self-renewal, and *Mllt6*, which has been implicated in leukemia progression [26–31]. The expression level of these selected genes alongside a gene-specific H4K16ac peak re-deposition was also changed in old CASIN-treated HSCs as in young samples (Fig. 2g, h and Additional file 1: Figure S2j-m). These data demonstrate that the H4K16ac mark might be linked to expression differences of selected genes, implying that specific changes in H4K16ac levels might indeed correlate to hematopoietic-relevant transcriptional changes, despite the lack of a statistically significant correlation between H4K16ac and gene expression in HSCs.

**Fig. 3:**
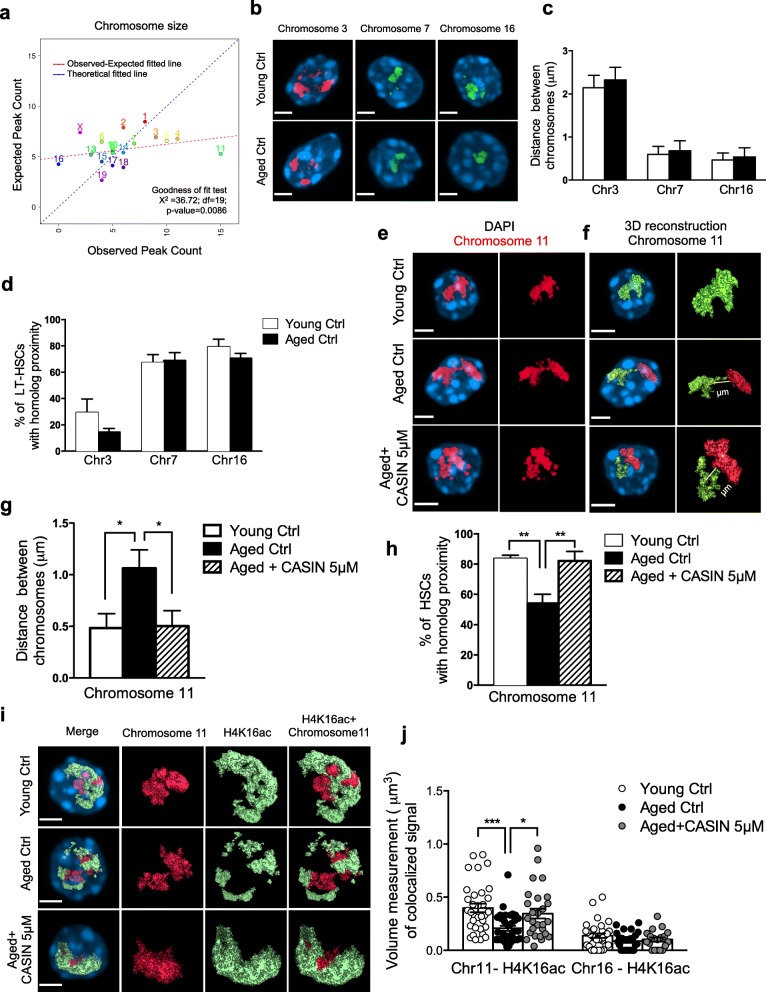
Changes in H4K16ac epipolarity are linked to the changes in chromosome 11 homologs distribution. **a** Comparison of the observed peak count and expected peak count relative to the chromosome size for the 118 peaks identified as described in panel M. Goodness of fit test was used, and it revealed that these 118 differentially bound H4K16ac peaks are not randomly distributed and chromosome 11 presented with the highest enrichment score. **b** Representative confocal 3D reconstruction of the nucleus (stained with DAPI, blue) and of chromosomes 3 (red), 7 (green), and 16 (green) by FISH stained with full-paint probe in young and aged HSCs. Bar = 2 μm. **c** Measurements of the distances between chromosomes 3 (*n* = 3 biological repeats, 28 cells for young, 21 cells for aged in total), 7 (*n* = 3 biological repeats, 25 cells for young, 26 cells for aged in total), and 16 (*n* = 3 biological repeats, 30 cells for young, 18 cells for aged in total) homologs in young and aged HSCs by Volocity 3D Image Analysis Software. **d** Percentage of young and aged HSCs with chromosome homolog proximity for chromosomes 3, 7, and 16, *n* = 3 biological repeats. **e** Representative confocal 3D reconstruction of the nucleus (stained with DAPI, blue) and of chromosome11 (red, FISH stained with a full-paint probe) in young, aged, and aged+CASIN 5 μM HSCs. Bar = 2 μm. **f** 3D reconstruction of the chromosome 11 homologs (red and green) and the distance measurement between them by Volocity 3D Image Analysis. In young HSCs, green color for two homologs shows that they are distributed very close to each other and are not recognized as two objects by the software. While in aged and aged+CASIN 5 μM HSCs, they are marked as two objects in green and red. Bar = 2 μm. **g** Measurements of the distances between chromosome 11 homologs by Volocity 3D Image Analysis. Shown are mean values + 1 SE; *p < 0.01; *n* = 6 biological repeats, 64 cells for young, 58 cells for aged, and 55 cells for aged+CASIN in total. **h** Percentage of young, aged, and aged+CASIN 5 μM HSCs with homolog proximity for chromosome11; **p < 0.01. **i** Representative confocal 3D reconstruction of young, aged, and aged+CASIN 5 μM HSCs after DNA-FISH+IF. H4K16ac (green), chromosome 11 (red), the nucleus is stained with DAPI (blue). Bar = 2 μm. **j** Volume measurement of the co-localized area between H4K16ac and chromosomes 11 and 16 by Volocity 3D Image Analysis; *p < 0.05, *n* = 3 biological repeats, 33 cells for young, 33 cells for aged, and 28 cells for aged+CASIN in total for chromosome 11 and 32 cells for young, 26 cells for aged, and 19 cells for aged+CASIN in total for chromosome 16

ChIP-seq analysis allowed us to define genomic regions, which bear different deposition (levels) of H4K16ac. However, this analysis does not inform on the 3D spatial distribution of H4K16ac peaks. The change in H4K16ac epipolarity (3D distribution) might not be due to differences in ChIP-seq mark deposition at a given genomic locus but might imply a non-random (re-)distribution (epipolarity) of the H4K16ac-associated genomic regions within the nucleus (see example depicted in Additional file 1: Figure S3a). While our ChIP-seq data demonstrate that changes in H4K16ac level at given loci are indeed detected upon HSC aging and rejuvenation, we also wanted to test whether a change in the position of H4K16ac-associated genomic regions might contribute to the change in epipolarity. To this end, next we tested whether H4K16ac is deposited at distinct genomic regions or whether it is randomly distributed. Therefore, we analyzed the observed peak count compared to the randomly expected distribution relative to chromosome size and to chromosome gene density. On a global scale, the 34,019 unique H4K16ac-associated peaks were primarily assigned to chromosomes 2, 7, and 11 (Additional file 1: Figure S3b). Interestingly, when considering only the 118 differential peaks common to young and CASIN-treated HSCs compared to aged HSCs (Fig. 2e), chromosome 11 presented with a very high non-random enrichment score, both relative to chromosome size (Fig. 3a) and gene density (Additional file 1: Figure S3c). Therefore, H4K16ac peaks common to young and CASIN-treated HSCs compared to aged HSCs are not randomly distributed but are selectively localized on chromosome 11.

Chromosomes are known to occupy distinct non-random territories in interphase nuclei [32]. The nature and position of territories are thought to play an important role in the maintenance of the appropriate nuclear architecture and nuclear compartmentalization necessary for proper cell function [20, 33, 34]. To test whether H4K16ac epipolarity might correlate with a distinct chromosome localization, we employed chromosome full-paint DNA-FISH staining of interphase nuclei of young, aged, and CASIN-treated HSCs to determine the relative 3D position of the distinct chromosome territories. For these analyses, HSCs were maintained ex vivo without growth factors for less than 16 h, and under these conditions, HSCs remain quiescent [16, 25]. Due to the high background generated by some of the probes, it was not possible to reliably analyze all chromosomes’ distribution (data not shown); however, we were able to determine the relative localization and homolog distance of chromosomes 3, 7, 11, and 16. While chromosome 3 homologs showed a relatively large distance to each other, and chromosome 7 and 16 homologs were close together, with no difference between young and aged HSCs (Fig. 3b–d). As there was little variation among individual cells in the distance of distinct chromosome homologs, and distinct homologs had distinct levels of distance, the data demonstrated that the distribution of at least some chromosomes in both aged and young HSCs is clearly not random. Chromosome 11 homologs also showed a non-random distribution and a close proximity within the nuclei of young HSCs. Interestingly, the distance of chromosome 11 homologs to each other did significantly increase in aged HSCs compared to young (Fig. 3e–h). Inhibition of Cdc42 activity (via CASIN) restored the proximity of chromosome 11 homologs in chronologically aged HSCs to the distance reported for young HSCs (Fig. 3e–h and Additional file 5: Video S1, Additional file 6: Video S2, Additional file 7: Video S3), demonstrating a causative role for the change in Cdc42 activity in regulating the position of chromosome 11 homologs in the nucleus of HSCs. Therefore, the data show that chromosome 11 homologs become more distant upon aging of HSCs, and Cdc42 activity controls the localization of chromosome territories, specifically chromosome 11, within the nucleus of HSCs.

To test whether the change in the position of chromosome 11 homologs might underlie the change in H4K16ac epipolarity in aged HSCs, we established DNA-FISH in combination with H4K16ac IF staining (3D-ImmunoFISH). The data revealed a high level of co-localization of chromosome 11 and H4K16ac in young and aged CASIN-treated HSCs (Fig. 3i, j; Additional file 1: Figure S3d and Additional file 8: Video S4, Additional file 9: Video S5, Additional file 10: Video S6), and both chromosome 11 homologs were close to each other in the H4K16ac-polarized compartment in young and CASIN-treated HSCs. In aged HSCs, H4K16ac was apolar and still co-localized with chromosome 11, but to a lesser extent. As anticipated by the low enrichment score (Fig. 3a and Additional file 1: Figure S3c), H4K16ac showed a very weak co-localization with chromosome 16 homologs that did not change in aged or aged CASIN-treated HSCs (Fig. 3j and Additional file 1: Figure S3e). In summary, the data imply selective changes in the position of chromosome 11 homologs, which are linked to the loss of H4K16ac epipolarity and the functional impairment of HSCs upon aging.

### Nuclear volume and nuclear shape are altered upon aging of HSCs

**Fig. 4:**
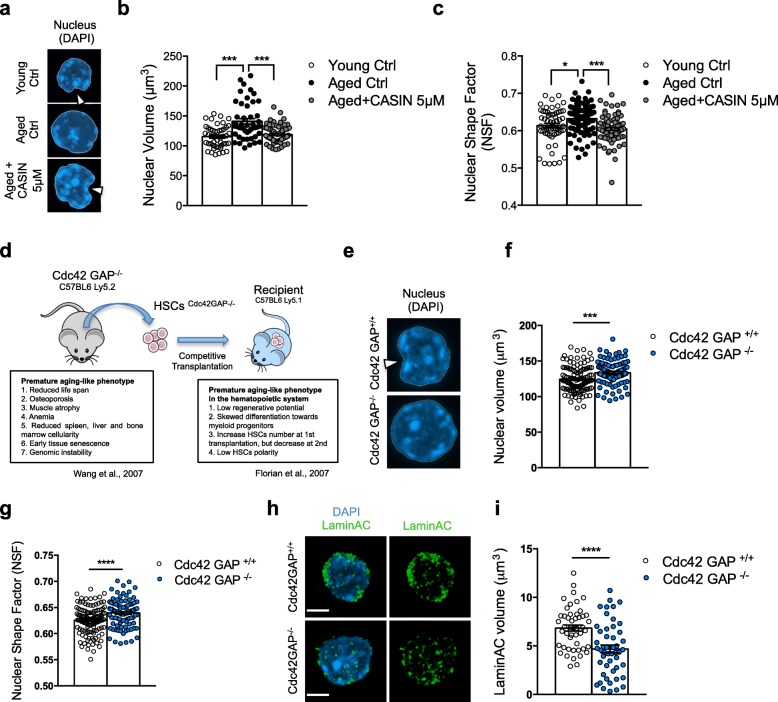
Nuclear volume and nuclear shape are altered upon aging of HSCs. **a** Representative confocal 3D reconstruction of the nucleus (stained with DAPI, blue) of young, aged, and aged+CASIN 5 μM HSCs. The arrowheads indicate the nuclear invagination normally observed in young and aged+CASIN HSCs. Bar = 2 μm. **b** Nuclear volume measurements by 3D-structured illumination (SIM) analysis (based on DAPI staining). ***p < 0.001; *n* = 3 biological repeats, 47 cells for young, 44 cells for aged, and 70 cells for aged+CASIN 5 μM in total. **c** Nuclear shape factor (NSF) measurements by Volocity 3D Image Analysis. The NSF scores the 3D shape of an object: 1 corresponds to a perfect sphere. *p < 0.05, ***p < 0.001; *n* = 6 biological repeats, 62 cells for young, 64 cells for aged, and 61 cells for aged+CASIN 5 μM in total. **d** Cartoon scheme summarizing premature aging-like phenotypes of Cdc42 GAP knockout (Cdc42GAP^−/−^) mice. **e** Representative confocal 3D reconstruction of the nucleus (stained with DAPI, blue) of Cdc42GAP^+/+^ and Cdc42GAP^−/−^ HSCs. The arrowheads indicate the nuclear invagination observed in Cdc42GAP^+/+^ HSCs. Bar = 2 μm. **f** Nuclear volume measurements by Volocity 3D Image Analysis (based on DAPI staining). ****p < 0.0001; *n* = 4 biological repeats, 119 cells for Cdc42GAP^+/+^, and 102 cells for Cdc42GAP^−/−^ mice in total. **g** Nuclear shape factor (NSF) measurements by Volocity 3D Image Analysis. The NSF scores the 3D shape of an object: 1 corresponds to a perfect sphere. **p < 0.01, *n* = 4 biological repeats, 118 cells for Cdc42GAP^+/+^, and 101 cells for Cdc42GAP^−/−^ mice in total. **h** Representative confocal 3D reconstruction of the nucleus (stained with DAPI, blue) and of LaminA/C (green) of Cdc42GAP^+/+^ and Cdc42GAP^−/−^ HSCs. Bar = 2 μm. **i** LaminA/C volume measurements by Volocity 3D Image Analysis (based on LaminA/C staining). **p < 0.01; *n* = 3 biological repeats, 50 cells for Cdc42GAP^+/+^, and 50 cells for Cdc42GAP^−/−^ mice in total

Our data demonstrate changes in chromosome 11 homolog distribution within the nucleus upon HSC aging, which also might be indicative of more general changes in the nuclear architecture of aged HSCs. We therefore determined the structure (volume and shape) of the nucleus of young, aged, and aged CASIN-treated HSCs. Both 3D confocal and 3D-structured illumination (3D-SIM) microscopy revealed that young HSCs have quite small and highly invaginated nuclei, while in contrast, nuclei of aged HSCs are significantly bigger and present with fewer invaginations. The nuclear invaginations in young HSCs form pocket-like structures (Fig. 4a–c and Additional file 1: Figure S4a). Upon CASIN treatment, nuclei of chronologically aged HSCs become smaller and highly invaginated, similar to the nuclei of young HSCs (Fig. 4a–c). To substantiate this finding by a genetic approach, we investigated the nuclei of HSCs sorted from Cdc42GAP^−/−^ mice, which present with the constitutively increased activity of Cdc42 and show premature aging of HSCs (Fig. 4d) [16, 35]. The nuclei of HSCs from chronologically young Cdc42GAP^−/−^ mice displayed an increased nuclear volume and a decrease in nuclear invaginations, similar to aged HSCs (Fig. 4e–g). Therefore, the shape and volume of HSC nuclei are altered upon aging, and these changes are regulated by Cdc42 activity. As a consequence, nuclear shape and volume can be targeted by CASIN treatment in chronologically aged HSCs.

Finally, we investigated the mechanism by which changes in Cdc42 activity might affect these distinct epigenetic aspects: H4K16ac epipolarity, chromosome 11 homolog proximity, and nuclear shape and volume which are altered upon aging of HSCs. We reasoned that structural molecules of the nuclear envelope, like nuclear lamins, might be downstream targets of changes in Cdc42 activity in HSCs. Nuclear lamins constitute the major intermediate filament proteins underlying the inner nuclear membrane and participate in many different nuclear architectural aspects and in genome maintenance by providing a scaffold for tethering chromatin and protein complexes that regulate genomic stability [36]. Lamins are divided into A/C- and B types based on their primary sequence homologies. A/C-type lamins are encoded by a single gene (*Lmna*). Mutations in the gene encoding LaminA/C, or factors affecting its maturation and dynamics, are also known to cause accelerated aging syndromes such as the Hutchinson-Gilford progeria syndrome [37]. HSCs sorted from Cdc42GAP^−/−^ mice, which present with constitutively increased Cdc42 activity and premature aging-like phenotypes, showed a significantly decreased level of LaminA/C (Fig. 4h, i), implying that Cdc42 activity plays a role in controlling LaminA/C expression in HSCs.

### LaminA/C expression in the hematopoietic system is specific to HSCs and is decreased upon aging

**Fig. 5:**
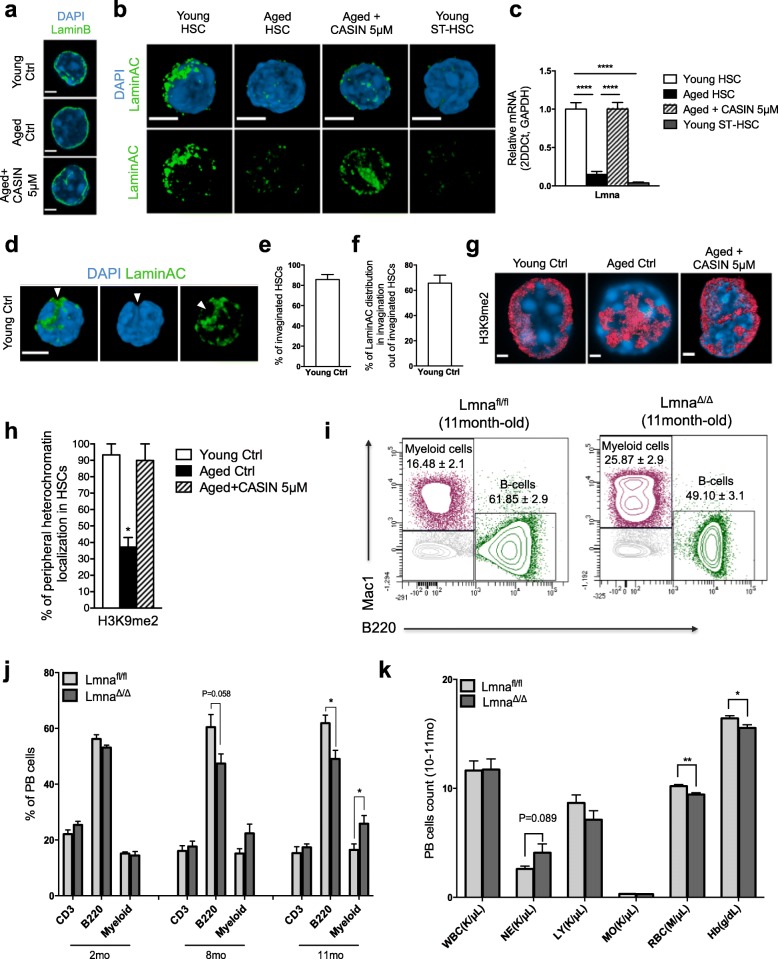
LaminA/C expression in the hematopoietic system is specific to HSCs and is decreased upon aging. **a** Representative confocal 3D reconstruction of the nucleus (stained with DAPI, blue) and LaminB (green) of young, aged, and aged+CASIN 5 μM HSCs. Bar = 2 μm. **b** Representative confocal 3D reconstruction of the nucleus (stained with DAPI, blue) and of LaminA/C (green) of young, aged, aged+CASIN 5 μM HSCs, and young ST-HSCs. Bar = 2 μm. **c** *Lmna* transcript levels in young, aged, aged+CASIN 5 μM HSCs, and in young ST-HSCs. Shown are mean values + 1 SE; *n* = 3 biological repeats; ****p < 0.0001. **d** Representative distribution of LaminA/C (green) in young HSCs. Bar = 2 μm. The arrow indicates LaminA/C distribution in the deep invagination of young HSCs nuclei. **e** Percentage of young HSCs with invaginated nuclei, *n* = 4 biological repeats, 45 single HSCs in total. **f** Percentage of young HSCs which have LaminA/C distribution in the invagination site of HSCs nuclei out of invaginated HSCs, *n* = 4 biological repeats, 45 single HSCs in total. **g** Representative confocal 3D reconstruction of the distribution of H3K9me2 in young, aged, and aged CASIN-treated LT-HSCs. Bar = 2 μm. **h** Percentage of young, aged, and aged+CASIN 5 μM HSCs presenting with peripheral heterochromatin localization of H3K9me2, *n* = 3 biological repeats, ~ 150 single HSCs for young and aged, 20 single HSCs for aged+CASIN 5 μM in total; *p < 0.05. **i** Representative FACS dot plots of B220^+^, CD3^+^, and myeloid (Gr1^+^, Mac1^+^, and Gr1^+^Mac1^+^) cells in control Lmna^fl/fl^ and Lmna^Δ/Δ/Vav-Cre^ PB. **j** Percentage of B220^+^, CD3^+^, and myeloid (Gr1^+^, Mac1^+^, and Gr1^+^Mac1^+^) cells in PB of control Lmna^fl/fl^ and Lmna^Δ/Δ/Vav-Cre^ mice; *p < 0.05, *n* = 4–6 mice. **k** Hematologic analyses of control Lmna^fl/fl^ and Lmna^Δ/Δ/Vav-Cre^ mice. *p < 0.05, **p < 0.01; *n* = 5–6 mice

A role for lamins in influencing the differentiation of blood cells was previously described [38], and changes in both LaminA/C and Lamin B are also known to affect chromatin [39–41]. Therefore, intrigued by the finding that Cdc42 activity regulates LaminA/C expression in HSCs, we further investigated lamins in young, aged, and aged CASIN-treated stem cells. IF analyses showed that Lamin B was localized to the nuclear periphery, with the pattern of distribution and the level of expression being almost identical in young, aged, and aged CASIN-treated HSCs (Fig. 5a). Interestingly, LaminA/C level was drastically reduced upon aging and restored to the level found in young HSCs upon inhibition of Cdc42 activity in aged HSCs, which is consistent with the increase in Cdc42 activity upon aging and with the reduction of LaminA/C expression in Cdc42GAP^−/−^ HSCs (Fig. 5b, c). Also, it was interesting to note that LaminA/C showed an overall dotted pattern of distribution specifically associated with the nuclear invaginations in young and aged CASIN-treated HSCs (Fig. 5d–f and Additional file 11: Video S7, Additional file 12: Video S8, Additional file 13: Video S9).

Consistent with the decrease of LaminA/C in aged HSCs, we also detected clear alterations in the nuclear compartmentation of lamin-associated domains (LADs) upon aging, as stained by H3K9me2 [42–44]. This repressive heterochromatin-associated histone PMT displayed marked reduction in its peripheral nuclear localization in aged stem cells (Additional file 1: Figure S5a), which was restored alongside the re-establishment of LaminA/C expression after CASIN treatment (Fig. 5g, h).

LaminA/C expression in young and aged ST-HSCs, as well as in LMPPs and committed myeloid progenitors (LK, lineage- c-kit+ sca1- BM cells; see Fig. 1b for the gating strategy), was significantly lower compared to HSCs (Additional file 1: Figure S5b), indicating a prominent role of LaminA/C for hematopoiesis primarily to the stem cell compartment. To further investigate the function of LaminA/C for hematopoiesis, we crossed *Lmna flox* mice with mice bearing the *Vav-Cre* transgene to delete LaminA/C specifically in the hematopoietic compartment (*Lmna*^Δ/Δ/Vav-Cre^) (Additional file 1: Figure S5c) [45, 46]. HSCs from young *Lmna*^Δ/Δ/Vav-Cre^ mice showed reduced levels of LaminA/C in HSCs but the knockout was incomplete, and we reproducibly detected residual LaminA/C protein (Additional file 1: Figure S5d-f). Nevertheless, hematopoiesis in these mice manifested with a decrease in B cell frequency, a reduced RBC count, a reduced concentration of hemoglobin in PB, an increase in myeloid cell frequency, and an elevated neutrophil count in peripheral blood (PB) as well as in BM already by 11 months of age (Fig. 5i–k and Additional file 1: Figure S5 g), consistent with a hematopoietic premature aging-like phenotype.

### The decrease of LaminA/C expression in HSCs determines changes in nuclear volume and shape, H4K16ac polarity, and chromosome 11 homologs proximity

**Fig. 6:**
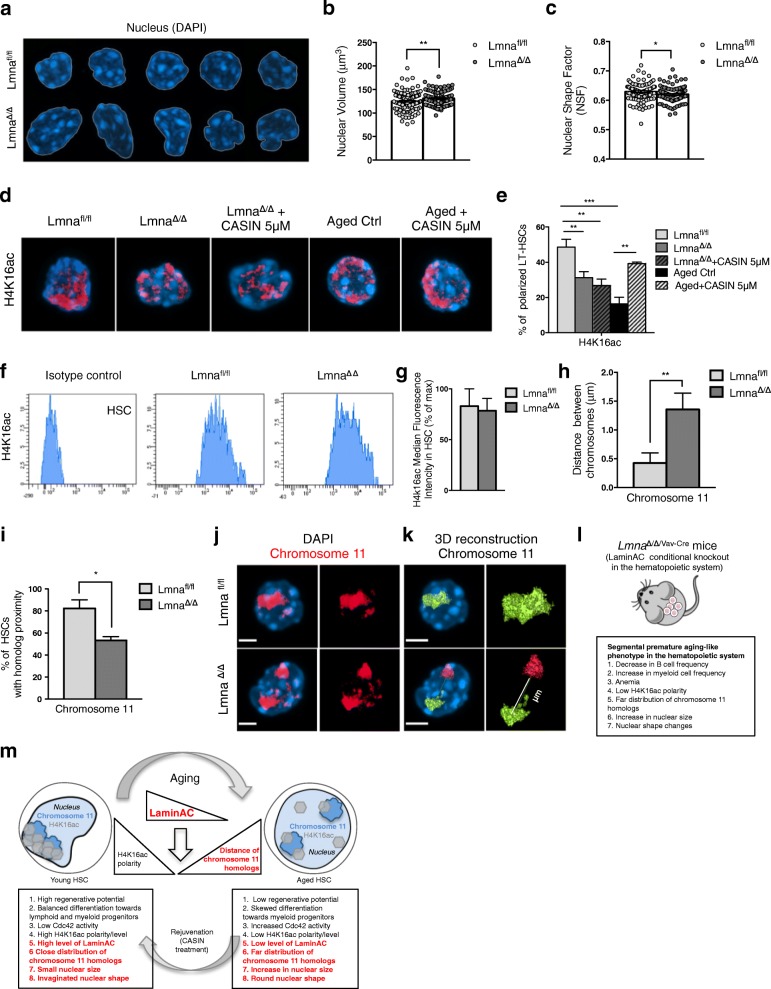
The decrease of LaminA/C expression in HSCs determines changes in nuclear volume and shape, H4K16ac polarity, and chromosome 11 homolog proximity. **a** Representative confocal 3D reconstruction of the nucleus (stained with DAPI, blue) of Lmna^fl/fl^ and Lmna^Δ/Δ/Vav-Cre^ HSCs. Bar = 2 μm. **b** Nuclear volume measurements by Volocity 3D Image Analysis (based on DAPI staining). **p < 0.01; *n* = 8 biological repeats; 100 single HSCs for Lmna^fl/fl^, 115 single HSCs for Lmna^Δ/Δ/Vav-Cre^ in total. **c** Nuclear shape factor (NSF) measurements. *p < 0.05; *n* = 4 biological repeats; 99 HSCs for Lmna^fl/fl^, 100 HSCs for Lmna^Δ/Δ/Vav-Cre^ in total. **d** Representative distribution of H4K16ac in Lmna^fl/fl^, Lmna^Δ/Δ/Vav-Cre^, Lmna^Δ/Δ/Vav-Cre^ + CASIN 5 μM, aged, and aged+CASIN 5 μM HSCs. Bar = 2 μm. Panels show DAPI (nucleus, blue), H4K16ac (red). **e** Percentage of Lmna^fl/fl^, Lmna^Δ/Δ/Vav-Cre^, Lmna^Δ/Δ/Vav-Cre^ + CASIN 5 μM, aged, and aged+CASIN 5 μM HSCs with a polar distribution of H4K16ac. Shown are mean values + 1 SE; *n* = 3–5 biological repeats, ~ 30–100 single HSCs scored per sample in total; *p < 0.05, ***p < 0.001. **f** Representative FACS histograms of an isotype control stained sample and of H4K16ac in Lmna^fl/fl^ and Lmna^Δ/Δ7Vav-Cre^ samples. HSCs gates are shown. **g** Median fluorescence intensity of H4K16ac in HSCs of control Lmna^fl/fl^ and Lmna^Δ/Δ/Vav-Cre^ mice; *n* = 3 biological repeats. **h** Measurements of the distances between chromosome 11 homologs in Lmna^fl/fl^ and Lmna^Δ/ΔVav-Cre^ HSCs by Volocity 3D Image Analysis; **p < 0.01; *n* = 3 biological repeats, 27 cells for Lmna^fl/fl^, 30 cells for Lmna^Δ/Δ/Vav-Cre^ in total. **i** Percentage of Lmna^fl/fl^ and Lmna^Δ/Δ/Vav-Cre^ HSCs with chromosome homologs proximity for chromosome 11; *n* = 3 biological repeats; *p < 0.05. **j** Representative confocal 3D reconstruction of the nucleus (stained with DAPI, blue) and of chromosome 11 (red, FISH stained with a full-paint probe) in Lmna^fl/fl^ and Lmna^Δ/Δ/Vav-Cre^ HSCs. Bar = 2 μm. **k** 3D reconstruction of the chromosome 11 homologs (red and green) and the distance measurement between them by Volocity 3D Image Analysis. In Lmna^fl/fl^ HSCs green, color for two homologs shows that they are distributed very close to each other and are not recognized as two objects by the software. While in Lmna^Δ/Δ/Vav-Cre^ HSCs, they are marked as two objects in green and red. Bar = 2 μm. **l** Summary of premature aging-like phenotypes in the hematopoietic system of LaminA/C conditional knockout mice. **m** Aging of HSCs compartment. Cartoon scheme integrating the novel main findings. The newly identified HSC aging phenotypes are highlighted in red

We further analyzed aging phenotypes in *Lmna*^Δ/Δ/Vav-Cre^ HSCs, and we detected larger and odd-shaped nuclei, similar to the nuclear shape of aged HSCs and distinct from wild-type young controls (Fig. 6a–c). Most importantly, young *Lmna*^Δ/Δ/Vav-Cre^ HSCs showed reduced H4K16ac polarity and increased nuclear volume that could not be rescued by CASIN treatment (Fig. 6d, e and Additional file 1: Figure S6a). These data strongly link LaminA/C expression, which is a downstream target of Cdc42 activity levels, to changes in H4K16ac epipolarity and nuclear volume. The absence of LaminA/C did not alter the overall amount of H4K16ac (Fig. 6f, g). Young *Lmna*^Δ/Δ/Vav-Cre^ HSCs consequently also showed an increase in the distance between chromosome11 homologs, similar to aged HSCs (Fig. 6h–k).

Collectively, these data demonstrate that LaminA/C is a downstream mediator of changes in Cdc42 activity in aged HSCs. LaminA/C regulates the nuclear shape and volume, H4K16ac epipolarity, and chromosome11 homolog proximity in HSCs upon aging, and consequently, low levels of LaminA/C confer aging-associated phenotypic and functional changes to HSCs (Fig. 6l). The data further confirm a role for changes in polarity of H4K16ac independent of changes in the level of this epigenetic mark, as loss of LaminA/C changes polarity, but not the level of H4K16ac. The data therefore imply at least a necessary contribution of changes in the nuclear architecture for the aging-associated changes in the function of HSCs.

## Conclusions

The contribution of the epigenetic drift to changes in the function of aged HSCs is currently the focus of intense debate. Recently, we and others showed that the function of HSCs corresponds to their epigenetic configuration but not always to their transcriptional state [47, 48], while epigenetic alterations in chromatin architecture were shown to precede transcriptional changes in somatic cell reprogramming [49]. These observations altogether support the concept of epigenetic and chromatin architecture changes as an instructive force for stem cell fate establishment that facilitates transcriptional changes in the differentiating progeny [50]. Our data show that the overall level and spatial distribution of multiple histone PTMs do not change to a large extent upon HSC aging, in agreement with the data by others [14, 15]. Aging of HSCs is thus more likely associated with a set of smaller local changes in the epigenetic make-up rather than a general global drift. Our data provide further support for a view in which changes in the level of a specific histone PTM (H4K16ac) per se do not affect HSC function upon aging, as increasing levels of histone acetylation in aged HSCs (like for example after NaB treatment, see Fig. 1k, n–t) do not rejuvenate stem cell function in transplantation assays. The fact that young *Lmna*^Δ/Δ/Vav-Cre^ HSCs present with a premature aging phenotype in the absence of a change in H4K16ac levels (see Fig. 6f, g) even support a model in which changes in the level of H4K16ac might actually be dispensable for conferring aging phenotypes on HSCs.

Interestingly, our findings underlie that chromatin and epigenetic architecture changes in aged stem cells are correlated to the functional alteration of HSCs upon aging and are reversible by targeting LaminA/C expression via Cdc42 activity. Indeed, we show that changes in the localization of at least chromosome 11 territories in aged HSCs are related to the shift from a polar to an apolar distribution of the H4K16ac mark upon aging and correlate to the functional impairments in aged and in *Lmna*^Δ/Δ/Vav-Cre^ HSCs. Pharmacological treatment with CASIN restores chromosome 11 architecture in aged HSCs and also improves old stem cell function in transplantation assays. To note, quantitative trait loci on chromosome 11 have been previously linked to aging in hematopoiesis as well as lifespan [51, 52]. Moreover, the frequency of HSCs in BM, known to be affected by chronological aging, is also associated with a locus on murine chromosome 11 [53].

In addition, here we identify the increase in nuclear volume and a change in the nuclear shape as novel phenotypes of aged HSCs. The genome forms an ordered, hierarchical structure that is believed, by orchestrating the nuclear architecture and the relative positions of chromosomes, to also orchestrate important cell biological functions [54–56]. Along with this line, our data provide a strong correlation between changes in nuclear volume and shape and in the position of specifically chromosome 11 homologs and changes in the function of HSCs. Our data also imply that these architectural changes are necessary and precede the alterations of HSC function upon aging, while their reversal is linked to HSC rejuvenation. However, the relationship between changes in the nuclear shape, nuclear volume, and chromosome 11 homolog position will need to be further investigated in a more stringent mechanistic approach.

Changes in nuclear volume and shape, changes in the distance of chromosome 11 homologs, and changes in H4K16ac epipolarity are a consequence of changes in LaminA/C expression. The reduction of LaminA/C in chronological young HSCs (*Lmna*^Δ/Δ/Vav-Cre^) consequently confers a segmental premature aging-like phenotype to HSCs. LaminA/C is known to be in close contact with large chromatin domains named lamina-associated domains (LADs). LADs are thought to help organize chromosomes inside the nucleus and have been associated with gene repression [42]. It has been reported that H3K9me2 and me3 have a prominent role in anchoring LADs to nuclear LaminA/C [57]. Consistent with the reduction in LaminA/C expression upon aging, we report here the alteration in H3K9me2 peripheral localization in aged HSCs while other reported a decrease in H3K9me3 [58], also supporting the overall alterations in the heterochromatin compartmentalization. As well, others recently described a role for LaminA/C in facilitating the functional organization of chromosomes, although not for stem cells or the aging of stem cells [59]. Alterations in LaminA/C in mesenchymal stem cells were also implicated in the deregulation of canonical Wnt/Notch signaling [60]. Interestingly, we previously reported an intrinsic autocrine switch from canonical to non-canonical Wnt signaling in HSCs upon aging that results in elevated activity of Cdc42 [25, 60]. We also demonstrate that the activity of Cdc42 controls the expression of LaminA/C in HSCs, since LaminA/C is increased in aged HSCs treated with CASIN (which lowers Cdc42 activity) and decreased in young Cdc42GAP^−/−^ HSCs, which present with constitutively increased activity of Cdc42 and a premature aging-like phenotype [16, 35]. Therefore, the data establish a Cdc42-LaminA/C regulation of the nuclear and chromatin architecture in HSCs. Importantly, in addition, we show here that chromatin architecture changes are reversible by changing levels of Cdc42 activity, revealing an unanticipated way to pharmacologically target and revert alterations of the epigenetic architecture in aged HSCs (Fig. 6m).

## Methods

### Mice

C57BL/6 mice (2–4 months old) were obtained from Janvier. Aged C57BL/6 mice (20–26 months old) were obtained from the internal divisional stock (derived from mice obtained from both The Jackson Laboratory and Janvier) as well as from NIA/Charles River. Congenic C57BL/6.SJL-*Ptprc*^*a*^/Boy (BoyJ) mice were obtained from Charles River Laboratories or from the internal divisional stock (derived from mice obtained from Charles River Laboratories). *Lmna*-floxed mice were kindly provided by Dr. C. Stewart [45]. To obtain the hematopoietic-specific knockout of LaminA/C, the *Lmna flox* mice were crossed with mice bearing the hematopoietic-specific *Vav-Cre* transgene (B6.Cg-Tg (Vav1-Cre) A2Kio/J). Genotyping of the Lmna^fl/fl^ and Lmna^Δ/Δ/Vav-Cre^ mice was conducted by a duplex PCR protocol. The conditional deletion was tested with primers LA-F1 CCAGCTTACAGAGCACCGAGCT, LA-F2 TCCTTGCAGTCCCTCTTGCATC, and LA-R1 AGGCACCATTGTCACAGGGTC and for the presence of Vav1-Cre transgene was used the following primers: Cre-For 5′ CTTCTAGGCCTGTACGGAAGTGTT 3′ and Cre-Rev 5′ CGCGCGCCTGAAGATATAGAAGAT 3′ [45, 46]. The effectiveness of the knock-out strategy was verified by intracellular flow cytometry and immunofluorescence staining in hematopoietic stem and progenitor cells. Antibodies used were anti-LaminAC (sc-20681), anti-LaminAC (sc-7292) from Santa Cruz Biotechnology, and anti-Lamin A+C (ab8984) from AbCam. And all showed only a knockdown of LaminA/C in HSCs, ranging from a reduction of 50 to 75% of the expression level. Cdc42GAP mice (9–11 months old) were obtained from CCHMC [35].

### Flow cytometry and cell sorting

PB and BM cell immunostaining was performed according to the standard procedures, and samples were analyzed on a LSRII flow cytometer (BD Biosciences). Monoclonal antibodies to Ly5.2 (clone 104, eBioscience) and Ly5.1 (clone A20, eBioscience) were used to distinguish donor from recipient and competitor cells. For PB and BM lineage analysis, the antibodies used were all from eBioscience: anti-CD3ε (clone 145-2C11), anti-B220 (clone RA3-6B2), anti-Mac-1 (clone M1/70), and anti-Gr-1 (clone RC57BL/6-8C5). Lineage FACS analysis data are plotted as the percentage of B220^+^, CD3^+^, and myeloid (Gr-1^+^, Mac-1^+^, and Gr-1^+^Mac-1^+^) cells among donor-derived cells in case of a transplantation experiment or among total white blood cells. As for early hematopoiesis analysis, mononuclear cells were isolated by low-density centrifugation (Histopaque 1083, Sigma) and stained with a cocktail of biotinylated lineage antibodies. Biotinylated antibodies used for lineage staining were all rat anti-mouse antibodies: anti-CD11b (clone M1/70), anti-B220 (clone RA3-6B2), anti-CD5 (clone 53-7.3), anti-Gr-1 (clone RB6-8C5), anti-Ter119, and anti-CD8a (clone 53-6.7) (all from eBioscience). After lineage depletion by magnetic separation (Dynalbeads, Invitrogen), cells were stained with anti-Sca-1 (clone D7) (eBioscience), anti-c-kit (clone 2B8) (eBioscience), anti-CD34 (clone RAM34) (eBioscience), anti-CD127 (clone A7R34) (eBioscience), anti-Flk-2 (clone A2F10) (eBioscience), and streptavidin (eBioscience). Early hematopoiesis FACS analysis data were plotted as a percentage of long-term hematopoietic stem cells (LT-HSCs, gated as LSK CD34^-/low^Flk2^−^), short-term hematopoietic stem cells (ST-HSCs, gated as LSK CD34^+^Flk2^−^), and lymphoid-primed multipotent progenitors (LMPPs, gated as LSK CD34^+^Flk2^+^) [61] distribution among donor-derived LSKs (Lin^neg^c-kit^+^sca-1^+^ cells). In order to isolate LT-HSCs, lineage depletion was performed to enrich for lineage-negative cells. Lineage-negative cells were then stained as aforementioned and sorted using a BD FACS Aria III (BD Bioscience). For intracellular flow cytometric staining, lineage-depleted young, aged, aged±CASIN 5 μM, and/or aged±NaB 5 mM BM cells were incubated for 16 h in IMDM+ 10% FBS at 37 °C, 5% CO_2_, and 3% O_2_. At the end of the treatment, the samples were moved on ice and stained again with the cocktail of biotinylated lineage antibodies. After washing, the samples were stained with anti-Sca-1 (clone D7) (eBioscience), anti-c-kit (clone 2B8) (eBioscience), anti-CD34 (clone RAM34) (eBioscience), anti-Flk-2 (clone A2F10) (eBioscience), and streptavidin (eBioscience). At the end of the surface staining, cells were fixed and permeabilized with Cytofix/Cytoperm Solution (BD Biosciences) and incubated with 10% donkey serum (Sigma) in BD Perm/Wash Buffer (BD Biosciences) for 30 min. Primary and secondary antibody incubations were performed at room temperature in BD Perm/Wash Buffer (BD Biosciences) for 1 h and 30 min, respectively. The secondary antibody for intracellular flow cytometry was a donkey anti-rabbit DyLight649 (BioLegend).

### HSC competitive transplantation

For competitive HSC transplantation, young (2–4-month-old) and aged (20–26-month-old) C57BL/6 mice (Ly5.2^+^) were used as donors. Two hundred HSCs were sorted into 96 multi-well plates and cultured for 16 h in HBSS+ 10% FBS±CASIN 5 μM or ±NaB (Sigma) 5 mM in a water-jacketed incubator at 37 °C, 5% CO_2_, and 3% O_2_. Stem cells were then mixed with 3 × 10^5^ BM cells from young (2–4-month-old) BoyJ competitor mice (Ly5.1^+^) and transplanted into BoyJ recipient mice (Ly5.1^+^). PB chimerism was determined by FACS analysis every 8 weeks up to 24 weeks post-primary transplants. The transplantation experiment was performed four times with a cohort of five recipient mice per group each transplant. In general, transplanted mice were regarded as engrafted when PB chimerism was higher or equal to 1.0%, and contribution was detected in all lineages.

### Immunofluorescence staining

Freshly sorted HSCs were seeded on fibronectin-coated glass coverslips. For polarity staining, HSCs were incubated 12–16 h in HBSS+ 10% FBS and when indicated treated with CASIN 5 μM or NaB 5 mM or left untreated. After incubation at 37 °C, 5% CO_2_, and 3% O_2_, in growth factor-free medium cells were fixed with BD Cytofix Fixation Buffer (BD Biosciences). After fixation, the cells were gently washed with PBS, permeabilized with 0.2% Triton X-100 (Sigma) in PBS for 20 min, and blocked with 10% donkey serum (Sigma) for 30 min. Primary and secondary antibody incubations were performed for 1 h at room temperature. Coverslips were mounted with ProLong Gold Antifade Reagent with or without DAPI (Invitrogen, Molecular Probes). Samples were imaged with an AxioObserver Z1 microscope (Zeiss) equipped with a × 63 PH objective. Images were analyzed with Zen software. Alternatively, samples were analyzed with an LSM710 confocal microscope (Zeiss) equipped with a × 63 objective. Primary raw data were imported into the Volocity Software package (Version 6.2, Perkin Elmer) for further processing and conversion into 3D images. As for polarity scoring, the localization of each single stained protein was considered polarized when a clear asymmetric distribution was visible by drawing a line across the middle of the cell or the nucleus. On average, a total of 20 to 150 HSCs were singularly analyzed per sample. Data are plotted as a percentage of the total number of cells scored per sample. The primary antibodies were as follows: anti-LaminB (sc-6217), anti-LaminAC (sc-20681), and anti-LaminAC (sc-7292) from Santa Cruz Biotechnology; anti-alpha tubulin (ab6160) and anti-Lamin A+C (ab8984) from AbCam; anti-H4K16ac (07-329) from Millipore; anti-H4K16ac (39168) from Active Motif; anti-H3K4me3 (ab8580), anti-H3K4me1 (ab8895), and anti-H3K27ac (ab4729) from AbCam; and anti-H4K8ac and anti-H4K5ac from Millipore. The secondary antibodies were anti-rat DyLight488-conjugated antibody, anti-rat DyLight647-conjugated antibody, anti-rabbit DyLight549-conjugated antibody, anti-rabbit DyLight488-conjugated antibody, and anti-mouse DyLight488-conjugated antibody (all obtained from Jackson ImmunoResearch).

### 3D DNA-FISH/Immuno-FISH (fluorescence in situ hybridization)

DNA and Immuno-FISH were done following the protocol by Chaumeil et al. [62]. Whole chromosome paint probes for chromosomes 3 and 11 (Spectrum Orange) and 7 and 16 (Spectrum Green) were generated and labeled according to Kosyakova et al. [63]. Cells were mounted with Vectashield antifade mounting medium with DAPI (Vector Laboratories) and scanned with a Zeiss LSM710 confocal microscope equipped with a × 63 objective. Z-stack images were acquired with 0.5 μm intervals and analyzed by Volocity 3D Image Analysis Software package (Version 6.2.0 Perkin Elmer).

### Distance and nuclear shape measurements

Nuclear shape measurement was done based on DAPI staining of nuclei and analyzed by Volocity 3D Image Analysis software. Nuclear shape factor (NSF) shows how similar is a 3D shape to a perfect circle. NSF is 1 for a perfect circle, and the circle becoming smaller for an irregular shape. “Measure distances” task was used to automatically measure the distance between two chromosomes from centroid to centroid. The centroid of the object is calculated by taking the average position of the voxels making up the object.

### 3D-structured illumination microscopy

For 3D-structured illumination microscopy (3D-SIM), a DeltaVision OMX V3 system (Applied Precision, GE Healthcare) was used, equipped with a × 100/1.40 NA Plan Apo oil immersion objective (Olympus, Tokyo, Japan), Cascade II:512 EMCCD cameras (Photometrics, Tucson, AZ, USA) and 405, 488, and 593 nm diode lasers. Image stacks were acquired with a *z*-distance of 125 nm and with 15 raw images per plane (five phases, three angles) resulting in an effective lateral and axial resolution of 120 nm and 300 nm, respectively. To obtain super-resolution image stacks, raw datasets were reconstructed using Wiener filter settings 0.004 and channel-specifically measured optical transfer functions (OTFs) using the softWoRx 6.0 software package (Applied Precision). To perform the analysis of nuclear volumes, 32-bit image stacks were shifted to positive values and converted to 16-bit composite TIFF stacks. Following, image stacks were imported into TANGO [64] using workflows as previously described [65]. Briefly, to generate nuclear masks, a watershed algorithm was used to segment signals from the DAPI channel, and morphological filters were applied to create coherent binary masks. The voxel coverage of these masks covering the entire nuclear volume was exported to Excel, and relevant distributions between different cell populations were plotted. Finally, to assess statistical significance, a two-tailed test was performed.

### Reverse transcriptase real-time PCR

Immediately after sorting, about 40,000 LT-HSCs from young and aged mice were lysed and processed for RNA extraction. RNA was extracted with the microRNA Extraction kit (Qiagen) and was used for cDNA conversion. cDNA was obtained and amplified with Ovation RNA Amplification System V2 (NuGEN). All real-time PCRs were run with TaqMan real-time PCR reagent and primers from Applied Biosystem on an ABI 9700HT real-time machine.

### Statistical analyses

Data were tested to meet normal distribution. The variance was similar between the groups that were statistically compared. All data are plotted as mean + 1 standard error (SEM) unless differently stated. The SEM is used to indicate the precision of an estimated mean. Such data representation does not affect the statistical analyses as variance information is utilized in the statistic tests. A paired Student’s *t* test was used to determine the significance of the difference between means of two groups. One-way ANOVA or two-way ANOVA were used to compare the means among three or more independent groups. Bonferroni post-test to compare all pair of dataset was determined when the overall *p* value was < 0.05. All statistical analyses were determined with Prism 6.0c version. In order to choose sample size, we used GraphPad StatMate Software Version 6.0b, estimating a standard deviation between 2 and 8 (depending on the experiment and the possibility of increasing sample size). For transplantation experiments, we estimated a sample size of 15 to 20 (assuming a standard deviation of 10 and a significant difference between means of at least 15). In transplantation experiments, samples were included in the analysis when engraftment was more or equal to 0.5% after 24 weeks, and contribution was detected in all lineages. Mice showing signs of sickness and with clear alterations of blood parameter and/or showing signs of major disease involving also non-hematopoietic tissues were excluded from the analysis. As for in vitro experiments, the samples were excluded from the analysis in case of clear technical problems (error in immune-blotting or staining procedures or technical problems with reagents). All criteria for exclusions of samples from in vivo or in vitro experiments were pre-established. In each figure, legend indicated the number (*n*) of biological repeats (samples obtained from experiments repeated in different days and starting from different mice) included in the final statistical analysis. Mice for experiments were randomly chosen from our in-house colonies or suppliers. All mice were C57/Bl6 females unless differently stated. The investigator was not blinded to the mouse group allocation nor when assessing the outcome (aged mice or young mice transplanted with aged BM stem cells require particular care and follow-up).

### Chromatin immunoprecipitation sequencing

Cells were fixed in 1% formalin for 10 min at room temperature, and fixation was stopped by adding glycine to a final concentration of 125 mM. After three washes with PBS, cell pellets were stored at − 80 °C. For sonication, cell pellets were resuspended in lysis buffer (1% SDS, 10 mM EDTA, 50 mM Tris, pH 8.1) supplemented with protease inhibitor cocktail (Roche) and processed in a Covaris S2 instrument using empirically established conditions that resulted in chromatin fragments between 150 and 300 bp in length (duty cycle 5%, intensity 4, cycles/burst 200; 40 cycles: 30 s on, 20 s off). Sonicated chromatin was cleared by centrifugation for 10 min at 13000 rpm. Ten percent of cleared chromatin was taken as input control. The H4K16ac antibody (Millipore) was coupled to protein A/G magnetic beads and washed × 2 with ChIP dilution buffer (0.01% SDS, 1.1% Triton X-100, 1.2 mM EDTA, 16.7 mM Tris-HCl, pH 8.1, 167 mM NaCl). Sonicated chromatin was first diluted 10-fold with ChIP dilution buffer, then the antibody-bead conjugates were added and incubated at 4 °C over night. Beads were first washed × 2 with Low Salt Immune Complex Wash Buffer (0.1% SDS, 1% Triton X-100, 2 mM EDTA, 20 mM Tris-HCl, pH 8.1, 150 mM NaCl) and then washed × 2 with LiCl Immune Complex Wash Buffer (0.25 M LiCl, 1% IGEPAL CA630, 1% deoxycholic acid (sodium salt), 1 mM EDTA, 10 mM Tris, pH 8.1). Then, precipitated chromatin was eluted and decrosslinked (2% SDS, 0.1 M NaHCO_3_, 0.25 M NaCl) for 4 h at 65 °C and afterwards treated with proteinase K for 1 h at 45 °C. DNA was purified using SPRI beads (Agencourt AMPure XP, Beckman Coulter) at a ratio of 1.8/1 (beads/DNA) and eluted in 15 μl ddH_2_O. Linear DNA amplification (LinDA) of ChIPed DNA was performed as described [66]. Briefly, ChIPed DNA was dephosphorylated for 10 min at 37 °C using 1 U TSAP (M9910, Promega) in NEB 4 buffer (B7004, NEB) followed by heat inactivation for 15 min at 75 °C. DNA was then *T* tailed for 20 min at 37 °C in the presence of 5 μM T-mix (92% dTTP, 8% ddCTP) and 0.25 mM CoCl_2_ using 20 U terminal transferase (M0252, NEB) followed by heat inactivation for 10 min at 70 °C. Next, 5 pmol T7-BpmI-oligo(A)_14_ primer (AATTAATACGACTCACTATAGGGCTGGAGAAAAAAAAAAAAAA) was annealed at 37 °C for 5 min and then double strand synthesis was performed for 55 min at 37 °C using 10 U Klenow fragment (M0210, NEB), 0.2 mM dNTPs. After heat inactivation, DNA was purified using SPRI beads at a ratio of 1.8/1 (beads/DNA) and eluted in 12.5 μl ddH_2_O. In vitro transcription was performed overnight using the RNAMaxx high yield kit (200339, Stratagene), and the resulting RNA was purified using SPRI beads (Agencourt AMPure XP, Beckman Coulter; beads/DNA ratio 1.8/1) followed by a reverse transcription (1 h, 42 °C; 10 min, 75 °C) using the T7-BpmI-oligo(A)_14_ primer and the Superscript III reverse transcription kit (18080044, Invitrogen). Second strand synthesis was performed using a custom reaction (20 μl first strand cDNA, 10 μl × 10 thermopol buffer, 1 ml × 100 BSA, 3 ml 10 mM dNTPs, 0.5 μl RnaseH, 1 μl Taq polymerase, 0.1 μl Pfu polymerase, 64.4 ml H_2_O) which was incubated in a thermocycler under the following conditions: 37 °C 5 min, 65 °C 1 min, 72 °C 30 min) followed by SPRI purification (beads/DNA ratio 1.8/1). Resulting double-stranded DNA was then digested with BpmI for 2 h at 37 °C, purified again with SPRI beads (beads/DNA ratio 1.5/1) and eluted in 13 μl ddH_2_O. Illumina sequencing libraries were prepared using the NEBNext Ultra DNA Library Prep Kit for Illumina (E7370, NEB) and the NEBNext Multiplex-Oligos for Illumina (E7335, NEB) following the manufacturer’s protocol. Since for all samples, the input amount of DNA was below 100 ng; the adaptors were diluted 1:10 in sterile water before adaptor ligation. Cleanup of adaptor-ligated DNA was performed without size selection using a 1.0/1 ratio of SPRI beads/DNA. For the PCR amplification of libraries, 0.5 μl of × 100 Syber Green was added to each reaction and the PCR amplification was performed on a LightCycler 480 (Roche) using the following cycling conditions: initial denaturation (98 °C, 30 s) followed by 9–12 cycles (98 °C, 10 s; 65 °C, 30 s; 72 °C, 30 s). The PCR reaction was stopped when the amplification curves had reached a fluorescence intensity of about 10. The PCR reactions were purified using SPRI beads at a ratio of 1.0/1 (beads/DNA). Sequencing libraries were multiplexed and subjected to 50 bp single-end sequencing on an Illumina HiSeq 2000 or HiSeq 2500 instrument (Illumina Inc., San Diego). ChIP-seq data can be accessed under Gene Expression Omnibus (GEO accession number: GSE120232 https://www.ncbi.nlm.nih.gov/geo/query/acc.cgi?acc=GSE120232).

### Sequence alignment and peak calling

Raw reads were trimmed using the trimLinDA script (available at http://www-igbmc.u-strasbg.fr/Gronemeyer) [67] mapped to the mouse reference genome (mm10) using the BWA aligner [68], and subsequently, duplicates and low-quality reads (MAQ score < 20) were removed using SAMtools [69]. For peak calling, generation of bedgraphs, differential binding analysis, and gene annotation, we used the Homer software package (http://homer.ucsd.edu/homer/download.html) [70]. The histone style parameters and the appropriate input sample were used to detect H4K16ac peaks.

### RNA-seq sample preparation and analysis

RNA-seq libraries were generated from 1000 pooled young, aged, and aged CASIN-treated HSCs (*n* = 4–5 biological repeats per sample) using Ovation® Single Cell RNA-Seq System (NuGEN Technologies Inc.). Manufacturer’s protocol was strictly followed. Briefly, reverse transcription was performed using oligo dT and selective primers to create first strand cDNA with randomly incorporated degradable nucleotide analog. This is degraded along with the RNA template in a downstream process, leaving single-stranded antisense cDNA fragmented to an average size of 230 nucleotides. Second strand cDNA priming was done using a forward adaptor with a random octamer attached to its 5′ end. After performing end repair, the free end of the newly synthesized double-stranded cDNA was ligated to the reverse adaptor and final enrichment PCR was performed. Samples were sequenced on the Illumina HiSeq2000 platform. The raw paired-end reads were adapter trimmed and quality filtered (phred score of > 20) using the cutadapt wrapper trim galore. Filtered sequences were aligned to the mouse genome mm10 RefSeq using TopHat and processed further using Cufflinks [71]. All downstream analysis including differential expression analysis, principal component analysis, between-group analysis, and additional statistical tests related to RNA-seq were performed using R and bioconductor packages [72] and in-house-developed scripts. The GSEA tool from broad MIT [73] was used for Gene Set Enrichment Analysis (GSEA). RNA-seq data have been deposited in NCBI’s Gene Expression Omnibus [74] and are accessible through GEO Series accession number GSE119466 (https://www.ncbi.nlm.nih.gov/geo/query/acc.cgi?acc=GSE119466).

## Additional files

Additional file 1: Supplementary **Figures S1-6.** (PDF 6151 kb)

Additional file 2: **Table S1.** 118 common genes that presented with differential binding of H4K16ac in young and aged CASIN-treated HSCs compared to aged control HSCs. (XLSX 49 kb)

Additional file 3: **Table S2.** RNA-seq expression profile of all genes analyzed (total genes 22548) in young, aged, and aged CASIN-treated HSCs. (XLSX 2254 kb)

Additional file 4: **Table S3.** Comparisons with three different stringency levels (no false discovery rate adjustment (no FDR) (no), FDR with Benjamini-Hochberg (medium), and FDR with Bonferroni adjustment (high) with 0.05 *p* value cutoff) of RNA-seq expression profile in young and aged CASIN-treated HSCs compared to aged control HSCs. (XLSX 112 kb)

Additional file 5: **Video S1.** 3D distribution of chromosome 11 (red) in young HSCs. Nucleus is stained with DAPI (blue). (AVI 16674 kb)

Additional file 6: **Video S2.** 3D distribution of chromosome 11 (red) in aged HSCs. Nucleus is stained with DAPI (blue). (AVI 15810 kb)

Additional file 7: **Video S3.** 3D distribution of chromosome 11 (red) in CASIN-treated aged HSCs. Nucleus is stained with DAPI (blue). (AVI 17096 kb)

Additional file 8: **Video S4.** 3D distribution of H4K16ac (green) and chromosome 11 (red) in young HSCs. Nucleus is stained with DAPI (blue). (AVI 18281 kb)

Additional file 9: **Video S5.** 3D distribution of H4K16ac (green) and chromosome 11 (red) in aged HSCs. Nucleus is stained with DAPI (blue). (AVI 19427 kb)

Additional file 10: **Video S6.** 3D distribution of H4K16ac (green) and chromosome 11 (red) in CASIN-treated aged HSCs. Nucleus is stained with DAPI (blue). (AVI 16314 kb)

Additional file 11: **Video S7.** 3D distribution of LaminA/C (green) in young HSCs. Nucleus is stained with DAPI (blue). (AVI 8653 kb)

Additional file 12: **Video S8.** 3D distribution of LaminA/C (green) in aged HSCs. Nucleus is stained with DAPI (blue). (AVI 7844 kb)

Additional file 13: **Video S9.** 3D distribution of LaminA/C (green) in CASIN-treated aged HSCs. Nucleus is stained with DAPI (blue). (AVI 9.9 MB) (AVI 9661 kb)

Additional file 14: Review history. (DOCX 48 kb)
