# Supplementary material for: LaminA/C regulates epigenetic and chromatin architecture changes upon aging of hematopoietic stem cells
Source: Genome Biol. 2018 Nov 7;19:189. doi: 10.1186/s13059-018-1557-3 (PMC6223039; doi:10.1186/s13059-018-1557-3)
Supplement: Supplementary file 1 — Supplementary Figures S1-6. (PDF 6151 kb) [file 13059_2018_1557_MOESM1_ESM.pdf]

Supplementary Figures

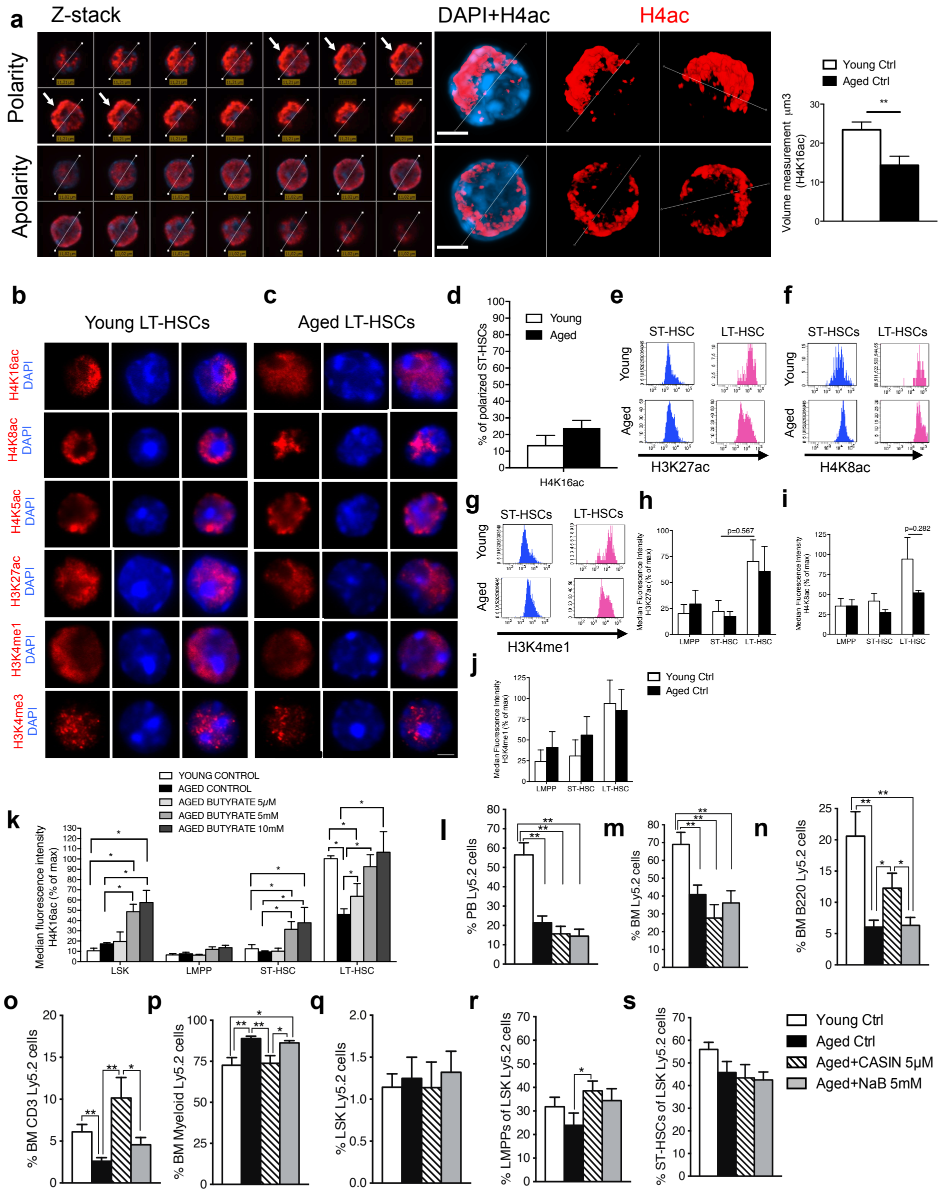

Figure S1. The change in the spatial distribution of H4K16ac upon aging is linked to a change in HSC function.
(a) Representative immunofluorescence z-stack and 3D reconstruction showing an epigenetic histone mark (H4ac) polar and apolar distribution within nuclei of HSCs. Arrowheads indicate signal distribution on the same side across the z-stacks of the nucleus. Lines, dividing the nucleus into two parts, indicate the polar or apolar distribution of the signal in 3D nuclei. Volume measurements of H4K16ac by Volocity 3D Image Analysis. Shown are mean values +1 S.E.; **p < 0.01.

(b-c) Representative IF staining of H4K16ac, H4K8ac, H4K5ac, H3K27ac, H3K4me1, H3K4me3 in young and aged HSCs. Nuclei are stained with DAPI (blue). Bar = 2μm.

(d) % of young and aged ST-HSCs with a polar distribution of H4K16ac, n=3-4 biological repeats; ~150-200 single cells scored per sample in total.

(e-g) Representative FACS histograms of H4K27ac (e), H4K8ac (f) and H3K4me1 (g) of young and aged samples. LT-HSCs and ST-HSCs gates are shown.

(h-j) Median fluorescence intensity of H4K27ac (h), H4K8ac (i) and H3K4me1 (j) plotted as % of young control in young and aged HSCs, ST-HSCs and LMPPs. Shown are mean values +1 S.E.; n=4-7 biological repeats; * $p < 0.05$.

(k) Median fluorescence intensity for H4K16ac plotted as % of young detected in young, aged and aged LT-HSCs, ST-HSCs, LMPPs and LSKs treated with NaB 5μM, 5mM and 10mM. Shown are mean values +1 S.E., n=3 biological repeats; *$p < 0.05$ vs young LT-HSCs.

(l) % of overall donor-derived Ly5.2$^+$ cells among Ly5.2$^+$ donor-derived cells in peripheral blood (PB) of recipient Ly5.1$^+$ mice. Shown are mean values +1 S.E., **$p < 0.01$; n=12-19.

(m-p) % of overall donor-derived Ly5.2$^+$ (m) and of B220$^+$ (n), CD3$^+$ (o) and myeloid (Gr1$^+$, Mac1$^+$ and Gr1$^+$Mac1$^+$) (p) cells among Ly5.2$^+$ donor-derived cells in BM of recipient Ly5.1$^+$ mice. Shown are mean values +1 S.E., *$p < 0.05$, **$p < 0.01$; n=12-19.

(q-s) % of LSKs among donor-derived Lin$^-$ Ly5.2$^+$ cells (q) and of LMPPs (r) and ST-HSCs (s) among donor-derived Ly5.2$^+$ LSKs in BM of recipient Ly5.1$^+$ mice. Shown are mean values +1 S.E., *$p < 0.05$; **$p < 0.01$; n=12-19.

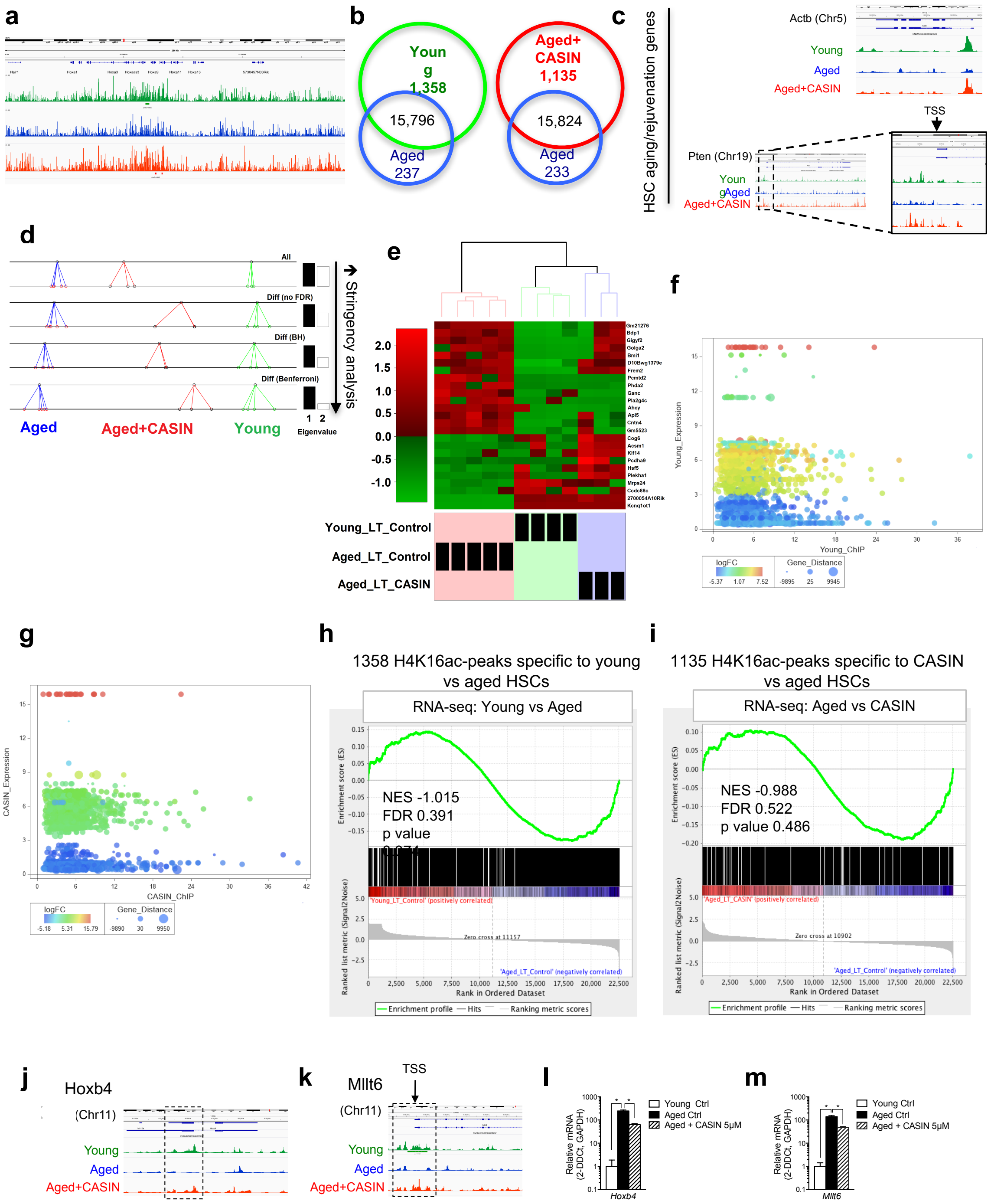

Figure S2. Changes in H4K16ac epipolarity are linked to changes in chromosome 11 homologs distribution

(a) General H4K16ac deposition ChIP-seq profile in young, aged and aged+CASIN 5μM HSCs.

(b) Venn diagrams showing 1358 H4K16ac peaks differentially enriched in young *vs* aged HSCs and 1135 different peaks when comparing CASIN *vs* aged samples.

(c) UCSC browser track showing H4K16ac signal for HSC aging/rejuvenation-related genes (such as *Pten* and *Actb*) in young, aged and aged+CASIN 5μM HSCs: n=3 biological repeats.

(d) Principal component based between group analysis (PCA) of RNA-seq data from young, aged and aged+CASIN HSCs. n=3-5 biological repeats. With increasing stringency of data filtering the distance between young and aged+CASIN HSCs becomes progressively smaller. Low stringency = list of differentially expressed genes without p-value adjustment; medium stringency = list of differentially expressed genes with either False Discovery Rate (FDR) or Benjamini-Hochberg (BH) adjusted p-value; high stringency = list of differentially expressed genes with Benferroni adjusted p-values.

(e) Heatmap clustering of RNA-seq data from young, aged and aged CASIN-treated HSCs based on differentially expressed genes comparing either young HSCs to aged HSCs or aged+CASIN HSCs to aged HSCs (Bonferroni adjusted p-value < 0.05).

(j) Correlation of overall RNA-seq data from young LT-HSCs (color code from blue to red according to log of fold change; on *y-axis*) to overall H4K16ac ChIP-seq data from young LT-HSCs (*x-axis*; size code indicates the distance of the peak from a given gene). No color distribution is seen according to the ChIP-seq profile.

(k) Correlation of overall RNA-seq data from aged CASIN-treated LT-HSCs (color code from blue to red according to log of fold change vs aged LT-HSCs; on *y-axis*) to overall H4K16ac ChIP-seq data from aged CASIN-treated LT-HSCs (*x-axis*; size code indicates the distance of the peak from a given gene). No color distribution is seen according to the ChIP-seq profile.

(h) Gene Set Enrichment Analysis (GSEA) for the 1358 chip-seq peaks different between young and aged HSCs in young vs aged RNA-seq data set.

(i) Gene Set Enrichment Analysis (GSEA) for the 1136 chip-seq peaks different between aged and CASIN HSCs in CASIN vs aged RNA-seq data set.

(j-m) UCSC browser track showing H4K16ac signal and real time PCR transcript level for *Hoxb4* (j and l) and *Mllt6* (k and m) in young, aged and aged+CASIN 5μM HSCs: n=3 biological repeats, * p < 0.0001.

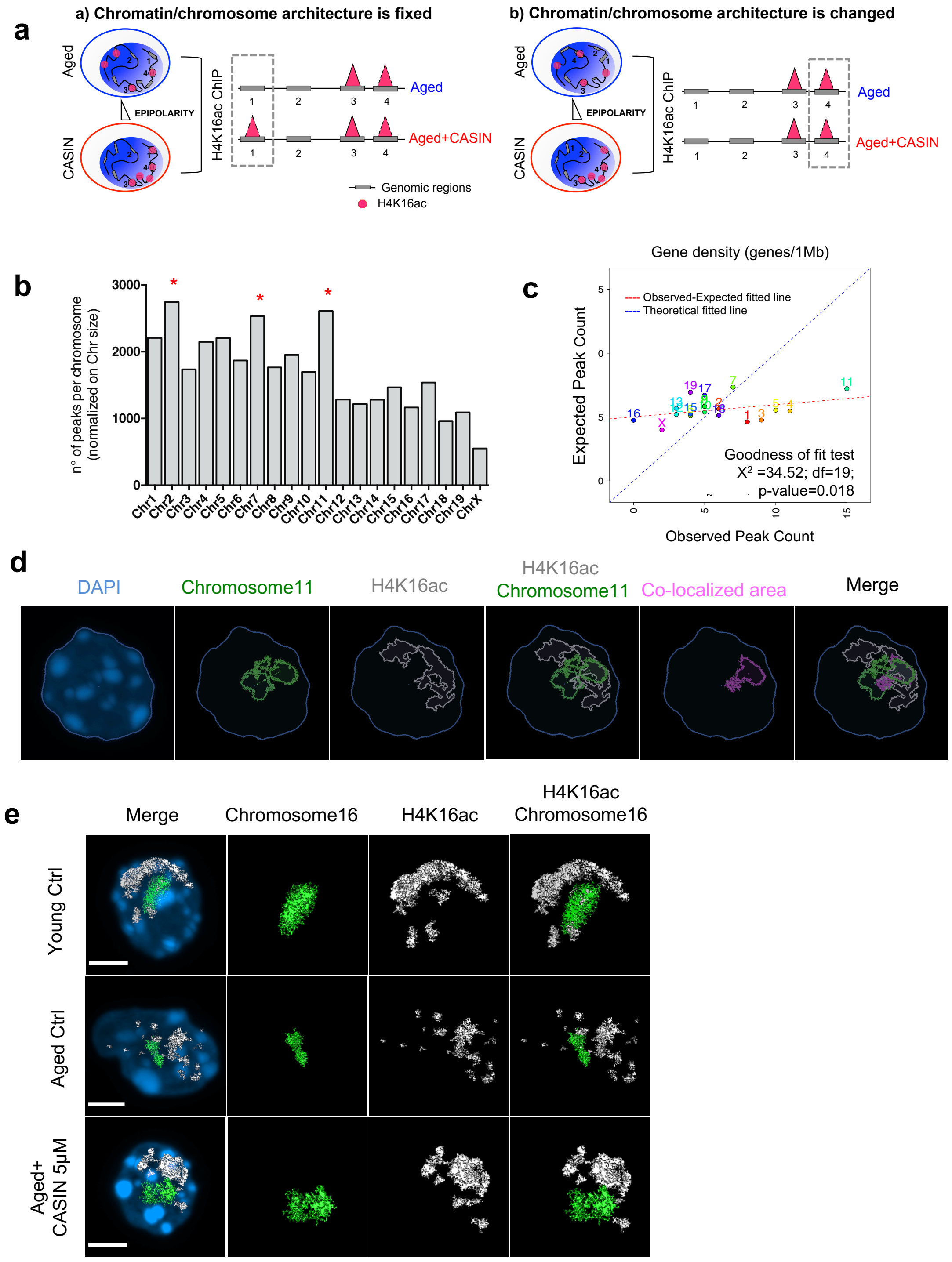

Figure S3. Changes in H4K16ac epipolarity are linked to changes in chromosome 11 homologs distribution

(a) Cartoon schemes of 2 different possibilities to explain epipolarity changes: H4K16ac different deposition and H4K16ac different architecture. (a) The chromatin/chromosome architecture within the nucleus is fixed. Hence the change in epipolarity from aged to CASIN-treated HSCs results in H4K16ac depositions and/or loss at given genomic

region. This means that the ChIP-seq profile will show differences at some specific gene loci, as for example at genomic region 1; (b) The chromatin/chromosome architecture within the nucleus is not fixed. The change in epipolarity from aged to CASIN-treated HSCs is contributed by H4K16ac-bound genomic regions that change their 3D organization. The ChIP-seq profile will not detect changes at these genomic regions as for example at genomic region 4. Therefore the change in epipolarity results in a reposition of specific genomic regions within the nucleus. To note one model does not exclude the other.

(b) Column graph showing the number of H4K16ac-associated peaks per chromosome normalized on chromosome size.

(c) Comparison of the observed peak count and expected peak count relative to the chromosome gene density for the 118 peaks identified as described in Figure 2 panel e. Goodness of fit test was used and it revealed that H4K16ac marks are not randomly distributed and chromosome 11 presented with the highest enrichment score.

(d) Reconstructed representative measurement of co-localized area of H4K16ac and chromosome 1 after DNA-FISH+IF. H4K16ac is shown in white, chromosome 11 in green, the nucleus is stained with DAPI (blue) and co-localized area is in pink. Reconstruction is done by Volocity 3D Image Analysis.

(e) Representative confocal 3D reconstruction of a young, aged and aged+CASIN 5μM HSC after DNA-FISH+IF. H4K16ac is shown in white, chromosome 16 in green and the nucleus is stained with DAPI (blue). The two chromosome16 homologs are barely stained by H4K16ac, independently from H4K16ac distribution. Bar = 2μm.

a

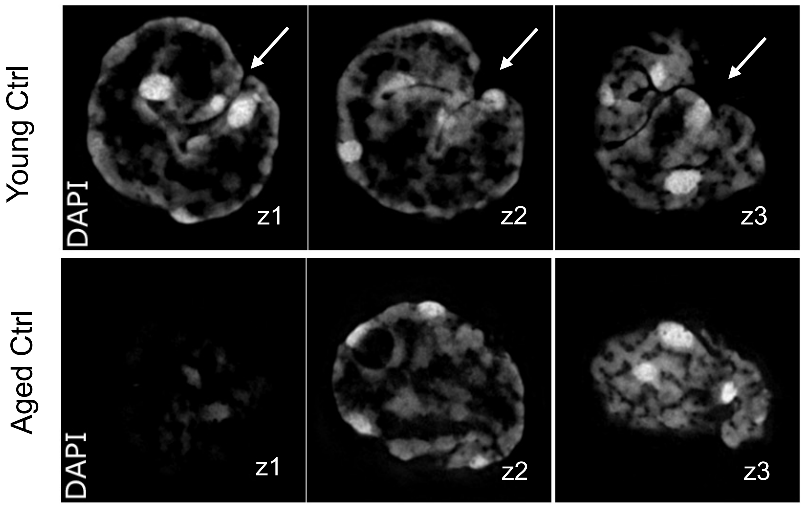

Figure S4. Nuclear volume and nuclear shape are altered upon aging of HSCs.

(a) Representative 3D-structured illumination (SIM) images of the nucleus (stained with DAPI, grey) of young and aged HSCs. The arrow indicates the deep invagination often seen in nuclei of young HSCs.

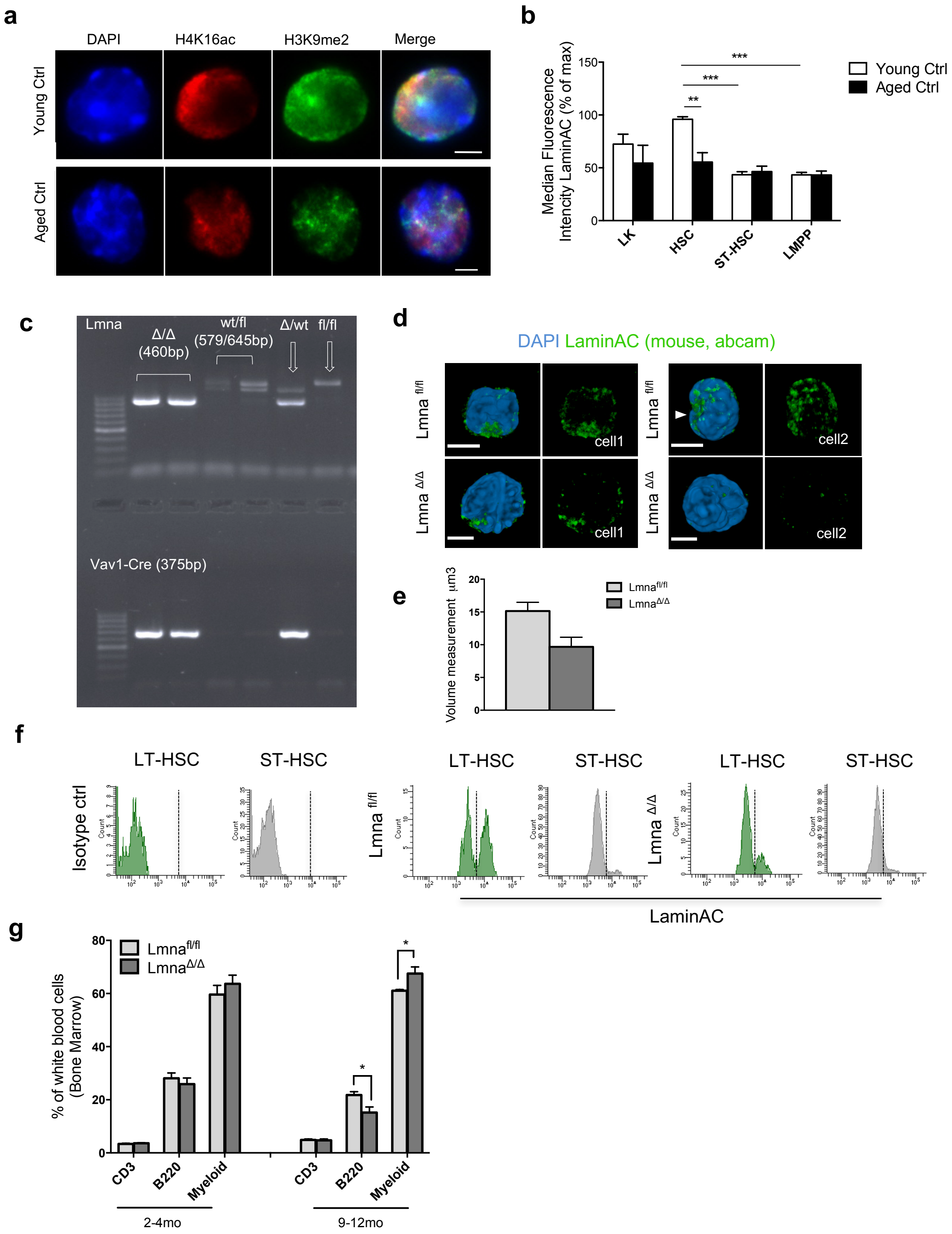

Figure S5. LaminA/C expression in the hematopoietic system is specific to HSCs and is decreased upon aging. **(a)** Representative IF staining of H4K16ac (red) and H3K9me2 (green) in young and aged HSCs. Nuclei are stained with DAPI (blue). Bar = 2μm.

(b) Median fluorescence intensity of LaminAC in LT-HSCs, ST-HSCs, LMPPs and LK cells of young and aged mice. Shown are mean values +1 S.E., **p<0.01, ***p < 0.001, n=4 biological repeats.

(c) PCR amplification of genomic DNA from peripheral blood of wild-type *Lmna* (579 bp), floxed *Lmna* (645 bp), null $Lmna^{\Delta}$ (460 bp) alleles and Cre allele (375bp).

(d) Representative distribution of LaminAC (green) in $Lmna^{fl/fl}$ and $Lmna^{\Delta/\Delta/Vav\text{-}Cre}$ HSCs. Bar = 2μm. The arrow indicates LaminAC distribution in the deep invagination of $Lmna^{fl/fl}$ HSCs nuclei. LaminAC antibody is a mouse monoclonal (ab8984).

(e) Volume measurements of LaminAC (ab8984) by Volocity 3D Image Analysis. Shown are mean values +1 S.E.; 10 single HSCs for $Lmna^{fl/fl}$, 10 single HSCs for $Lmna^{\Delta/\Delta/Vav\text{-}Cre}$ in total.

(f) Representative FACS histograms of LaminAC for $Lmna^{fl/fl}$ and $Lmna^{\Delta/\Delta/Vav\text{-}Cre}$ HSCs and ST-HSCs**.** LaminAC antibody is a rabbit polyclonal (sc-20681).

(g) % of $B220^+$**,** $CD3^+$, and myeloid ($Gr1^+$, $Mac1^+$ and $Gr1^+Mac1^+$) cells in BM of control $Lmna^{fl/fl}$ and $Lmna^{\Delta/\Delta/Vav\text{-}Cre}$ mice; * p < 0.05, n=12mice 2-4month-old, n=5mice 9-12month-old.

a

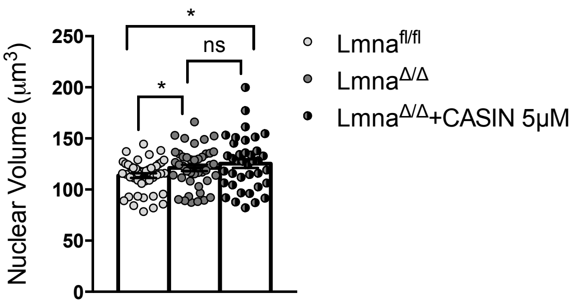

Figure S6. The decrease of LaminA/C expression in HSCs determines changes in nuclear volume and shape, H4K16ac polarity and chromosome 11 homologs proximity.

(a) Nuclear volume measurements by Volocity 3D Image Analysis (based on DAPI staining). ** p < 0.01; n=3 biological repeats; 46 single HSCs for $Lmna^{fl/fl}$, 42 single HSCs for $Lmna^{\Delta/\Delta/Vav\text{-}Cre}$, 38 cells (to be checked) $Lmna^{\Delta/\Delta/Vav\text{-}Cre}$ + CASIN 5 in total.
