# Supplementary material for: LaminA/C regulates epigenetic and chromatin architecture changes upon aging of hematopoietic stem cells
Source: Genome Biol. 2018 Nov 7;19:189. doi: 10.1186/s13059-018-1557-3 (PMC6223039; doi:10.1186/s13059-018-1557-3)
Supplement: Supplementary file 14 — Review history. (DOCX 48 kb) [file 13059_2018_1557_MOESM14_ESM.docx]

**First round of review**

**Reviewer 1**

**Are the methods appropriate to the aims of the study, are they well described, and are necessary controls included?**
Yes.

**Are the conclusions adequately supported by the data shown?**

Yes, with the exceptions outlined below

**Are sufficient details provided to allow replication and comparison with related analyses that may have been performed?**

Yes.

**Does the work represent a significant advance over previously published studies? Please explain.**

Yes. This paper is a follow-up study of Florian et al. 2012 where the authors showed a correlation between CDC42 activity and the loss of intra-nuclear polarity as indicated by the specific location of histone H4K16 acetylation. In this paper they show that CDC42 regulates LaminA/C expression and that correct organization of the nuclear lamina is important for polarization. These are important results as it indicates stem cell function requires a specific nuclear shape and organization mediated by Lamins.

**Is the paper of broad interest to others in the field, or of outstanding interest to a broad audience of biologists?**

Yes. The results are of general interest for the stem cell field and beyond.

**Comments to authors**:

This paper is a follow-up study of Florian et al. 2012 where the authors showed a correlation between CDC42 activity and the loss of intra-nuclear polarity as indicated by the specific location of histone H4 K16 acetylation. In the manuscript here, the authors show that (i) not all histone modifications show extensive polarization and that loss of H4K16Ac is specific to old HSCs, (ii) elevated levels of H4K16Ac are insufficient to restore polarity in old HSCs and for the restoration of HSC function, (iii) other chromatin architectural features such as Chr11 allelic distance and nuclear shape are associated with ageing and depolarization, and finally (iv) that high CDC42 activity correlates with the repression of LaminA/C expression and the loss of the epipolarity/ageing phenotype, analysing cells from the respective knock-out mice. The conclusions are sound and are underpinned by a large number of experiments which are mostly of excellent quality (with the exceptions outlined below). The majority of these experiments are of a descriptive nature, but at a very high level as the authors are using different types of manipulations (i.e. knock-outs) to infer cause and effects.

However, the manuscript cannot be published in its current form, as it lacks focus and jumps around and could seriously benefit from editing by a native speaker. I am also not entirely convinced that the author is on top of the Lamin literature.

Major comments:

1. The paper appears to have been written in a "historical" fashion, meaning that the authors appeared to have favoured the hypothesis that H4K16Ac on specific genes was responsible for polarity. Figure 1 debunks this idea, but why is the analysis of the ChIP experiments in the paper? It is fine to show that H4K16Ac levels as measured by ChIP are much lower in old cells, Casin treated or not, but the attempt to peak call and identify differentially acetylated genes really does not add anything to the manuscript. The quality of the data with such small numbers of cells is not great and doing Venn diagrams is not a good idea. Moreover, calling and comparing peaks makes no sense in the absence of a gene expression profile and I am surprised that the authors did not perform such a comparison as RNA-Seq does not require more cells than ChIP Seq. Just remove this part and only show that heatmaps (as outlined below) and a handful of screenshots to highlight data quality.

2. I do not see the point in plotting the tag counts around the TSS (Fig 2b) when the majority of the signal is in intronic and intergenic regions. I would suggest to plot two groups: One around the TSS (as shown) and one at all other regions centred on the centre of the peak/acetylated region. The latter will look slightly untidy as the "peaks" are very broad, but will prove the point that acetylation levels are lower in chromatin where it matters. 

3. It would make more sense to describe the changes in nuclear shape and volume before describing the distances between chromosomes. Why the authors look at Chr 11 (Fig 2 g is seriously badly described) - would it not make more sense to already introduce Chr11 as the one that appears to carry ageing - related genes? We only learn this fact in the discussion.

4. The Lamin studies are interesting and establish the axis CDC42 - Lamin - polarity, but they could be driven further. The manuscript entirely ignores the literature of Lamin-associated domains (LADs, see this review as a good primer into the field: doi: 10.1016/j.cell.2017.04.022). 
LADs are associated with repressive chromatin and from this manuscript it appears as if such an association is important for HSC polarity (and quiescence?). From the DAPI stain it is already clear that the H4K16Ac staining is distinct form the more dense heterochromatic regions which should contain LADs. The relationship between the two compartments should therefore be better highlighted using additional immunofluorescence experiments.

5. The discussion/conclusions should be focussed on the main points as I summarize above and discuss the Lamin literature in detail and not outline why some experiments cannot be done - I agree with all that is said but that is the subject of a rebuttal letter

6. The abstract is really sloppily written and does not really summarize the findings. It needs restructuring, maybe along the lines of my summary above?

Minor points:

1. The Figures are awfully dense. I don't know what the actual format is, but I would suggest to generate more figures and/or move some of the controls into the Supplements. I also seriously dislike it when a sentence is interrupted by a lengthy description of what is in the supplements - see page 5, line 86 - 89). Phrase properly.

2. Fig. 1 o - t: the legend of which bar describes what is tucked onto 1t, but is relevant for all panels - except s. Please place underneath o - r (horizontally)

3. The manuscript contains a multitude of grammatical errors and funny sentences, examples are:
Abstract "Here, we show that changes in H4K16ac upon aging and rejuvenation of HSCs are underlay to a great extent by a shift of chromosome 11 architecture.." 
Background section: For example, it was reported an emergence 54 of bivalent domains marked by both H3K4me3 and H3K27me3 in aged HSCs compared to young [14]
And so on. Please ask a native speaker to go through the text

I hope that the author finds my comments helpful

**Reviewer 2**

**Are the methods appropriate to the aims of the study, are they well described, and are necessary controls included?**
Yes, but improvements can be made.

**Are the conclusions adequately supported by the data shown?**

Many are, but not all. Please see detailed critique.

**Are sufficient details provided to allow replication and comparison with related analyses that may have been performed?**

Additional information on certain experiments needed.

**Does the work represent a significant advance over previously published studies? Please explain.**

If noted issues are addressed, yes.

**Is the paper of broad interest to others in the field, or of outstanding interest to a broad audience of biologists?**

Yes. Yes, because we do not understand the mechanisms of aging.

**Comments to authors:**

Grigoryan et al report that HSCs exhibit polar distribution of H4K16Ac in young but not old mice; that PTM polarity is rare; that CASIN (a Cdc42 inhibitor), but not NaB, treatment, of aged HSCs restores H4K16Ac polarity, and that this polarity is stably inherited after transplantation such that the CASIN-treated old HSCs reconstitute hematopoiesis similar to young HSCs and have H4K16Ac polarity after transplant. A link specifically to chromosome 11 is reported. The authors investigate nuclear shape and volume, observing larger nuclei with fewer invaginations upon HSC aging, which again were rescued upon treating old HSCs with CASIN. These findings suggest that more general changes in nuclear structure occur during aging, so the authors explored nuclear lamins as potential mediators of the aging phenotypes. LaminA/C, but not LaminB, deletion caused changes in nuclear architecture and a pre-aging-like phenotype. These are interesting findings that build upon the group's previous work. The authors make a considerable effort to characterize several nuclear and chromatin parameters in young and old HSCs and provide evidence for several selective changes with age. I agree with the authors' assertion that it is difficult (impossible?) to directly manipulate chromosome localization. In the end, though, because most changes are correlative, I have a difficult time understanding the causal relationships or how it is even possible that two sister chromosomes are co-localized to one side of the nucleus selectively in young HSCs and how this is affected via Cdc42-mediated changes to H4K16Ac. 

Specific points and suggestions:
* P 7: The 3 patterns described do not make sense since H3K27Ac is not significantly different between HSCs and more differentiated cells (Figure 1h). 
* Please be consistent with the histone modification labeling; Figure S1b uses "Me1H3K4" whereas S1c uses "H3K4Me1", etc.
* Figure 1j, k; Figure S1k: preferable to use brackets (as in Figure 1o,p,q,r, etc) to indicate which comparisons are different. 
* P 9: "distribution of H4K16Ac is at least necessary…" needs to be rephrased. One cannot conclude, at this point, that H4K16Ac repolarization is necessary, and the sentence is awkwardly phrased, as are several others. The conclusion "..epipolarity in young HSCs and its loss upon aging is definitely not common among histone PTMs…" and yet the author's report changes in epipolarity, albeit at a smaller extent compared to H4K16Ac, in 4 out of 6 histone modifications assayed. It may be better to describe the H4K16Ac changes as "selective" rather than "specific".
* If the epipolarity changes associated with aging are primarily manifested on chromosome 11, how do the authors envision that this results in global (involving many/all additional chromosomes) changes in (H4K16Ac) epipolarity, nuclear shape and volume, etc, and in a CASIN-mediated "rejuvenation" phenotype? 
* P9: please describe the sample preparation for ChIPseq more clearly: were all samples incubated in vitro? Was a comparison to freshly isolated cells made? Additionally, given the very large difference in overall signal intensity between young and old in Figure 2b, it is counterintuitive that only ~1300 differential peaks were found between young and aged, and that as many as ~1100 were found in aged versus aged+CASIN treated samples where the signal intensities are very similar. Please explain. 
* P 9: What does it mean that genes linked to both self-renewal, differentiation, and leukemogenesis were identified by ChIPseq? Would it not make sense if either self-renewal or differentiation were enriched, but not both? The observation of one or a couple of genes (Figure 2f) that are both differential by ChIPseq and by expression does not substantiate the conclusion that "H4K16Ac marks are linked to expression differences". Which loci are increasing in H4K16Ac binding after NaB treatment? Is this specific to CASIN treatment that when comparing CASIN to Old mice? The conclusions made from the ChIPseq and expression data in Figure 2 should be substantiated by more global, objective analyses rather than based on a few randomly selected genes. Identifying the genes that are unique to CASIN treatment when compared to NaB treatment might narrow down the mechanism of apparent regeneration after CASIN treatment.
* Figure 2h and many others lack information on independent biological repeats, and some that do have this information have fewer than 3 independent repeats. Most, if not all, conclusions should be based on results from at least three independent experiments, and this should be clearly indicated in the legends. 
* P 9: why was DNA-FISH performed on cells maintained ex vivo rather than on freshly isolated cells? Was a comparison made between cultured and fresh cells? 
* P 10: The conclusion, "These data demonstrate that H4K16Ac marks are linked to expression differences of gene loci important for hematopoiesis, implying that changes in specific gene loci…" is a stretch as only 3 genes were probed for expression differences. A more in-depth analysis of expression differences and H4K16Ac differences in genes specific to HSC function would strengthen the conclusion made.
* Is Cdc42 inhibition known to alter gene expression and are the expression profiles revealed here similar to previous report? Cdc42 has been reported to regulate a range of HSC activities (migration, proliferation, etc): how does that fit with the epipolarity findings described here?
* Figure 3a-c: same questions as posed above for ChIPseq samples: were all samples incubated in vitro? Was a comparison to freshly isolated cells made? 
* Figure 4b: can the authors implement a quantitative measure that substantiates the statement that the LaminA/C preferentially localize near nuclear invaginations?
* The link between LaminA/C deficiency and aging is a bit tenuous. Have the authors investigated the aging phenotype in LaminA/C-/- mice older than 11 months (18-24 months)? Is the phenotype progressively worse? Have LaminA/C-/- HSCs from aged mice been transplanted to test functionality? If the authors have such experiments started or mice of age available, the results could potentially add significant weight to the current conclusions. 
* The authors conclude that nuclear structure contributes to the aging phenotype, but the link to Cdc42 was not clear. Was the expression level or protein level of Cdc42 measured in the Lamin A knockout? Does CASIN treatment rescue the phenotype in the Lamin A knockout animals? Is there an additive effect if both Lamin A and Cdc42 were knocked out? Can the aging defects be rescued by restoring the expression of LaminA/C to young levels? 
* Figure 5: Some clarification as to why Tubulin distribution was investigated and interpretation of the results observed in the Lamin A/C knockout are needed.

**Authors’ response to reviewers**

*Reviewer #1:*

*This paper is a follow-up study of Florian et al. 2012 where the authors showed a correlation between CDC42 activity and the loss of intra-nuclear polarity as indicated by the specific location of histone H4 K16 acetylation. In the manuscript here, the authors show that (i) not all histone modifications show extensive polarization and that loss of H4K16Ac is specific to old HSCs, (ii) elevated levels of H4K16Ac are insufficient to restore polarity in old HSCs and for the restoration of HSC function, (iii) other chromatin architectural features such as Chr11 allelic distance and nuclear shape are associated with ageing and depolarization, and finally (iv) that high CDC42 activity correlates with the repression of LaminA/C expression and the loss of the epipolarity/ageing phenotype, analysing cells from the respective knock-out mice. The conclusions are sound and are underpinned by a large number of experiments which are mostly of excellent quality (with the exceptions outlined below). The majority of these experiments are of a descriptive nature, but at a very high level as the authors are using different types of manipulations (i.e. knock-outs) to infer cause and effects. However, the manuscript cannot be published in its current form, as it lacks focus and jumps around and could seriously benefit from editing by a native speaker. I am also not entirely convinced that the author is on top of the Lamin literature.*

**We thank the referee for the time he/she spent on commenting on our manuscript. We are glad that the referee found our manuscript interesting and our experiments mostly of excellent quality. We addressed his/her suggestions in this revised version. In detail, we provide now RNA-seq data to support the analysis of the H4K16ac ChIP-seq data. Moreover, we include novel data on lamin associated domains (LADs) by staining young and old HSCs with the heterochromatin marker H3K9me2.**

**Below our point-to-point response to the comments:**

*Major comments:*

*1. The paper appears to have been written in a "historical" fashion, meaning that the authors appeared to have favoured the hypothesis that H4K16Ac on specific genes was responsible for polarity. Figure 1 debunks this idea, but why is the analysis of the ChIP experiments in the paper? It is fine to show that H4K16Ac levels as measured by ChIP are much lower in old cells, Casin treated or not, but the attempt to peak call and identify differentially acetylated genes really does not add anything to the manuscript. The quality of the data with such small numbers of cells is not great and doing Venn diagrams is not a good idea. Moreover, calling and comparing peaks makes no sense in the absence of a gene expression profile and I am surprised that the authors did not perform such a comparison as RNA-Seq does not require more cells than ChIP Seq. Just remove this part and only show that heatmaps (as outlined below) and a handful of screenshots to highlight data quality.*

**We have generated RNA-seq data from young, old and old CASIN-treated HSCs and we include these data set in the revised version of the manuscript (Fig 2a and f and Fig. S2d-i). As suggested by the referee, we have investigated the transcriptome of HSCs for the expression level of H4K16ac peak-associated genes. Gene set enrichment analyses (GSEA) showed no significant enrichment. We conclude that there is no correlation between gene expression (both for overall expression and differentially expressed genes) and H4K16ac**

**deposition in young and aged CASIN-treated HSCs compared to aged HSCs. Please see lines 149-150 and lines 159-179. We agree with the referee that the ChIP-seq dataset mainly confirms a strong reduction of**

**H4K16ac deposition in aged HSCs (in agreement with the quantifications by immunofluorescence analyses as well as intracellular flow cytometry; Fig. 1g and j and Fig. S1a). However, even if most of the H4K16ac associated genomic regions are not at TSS or promoters (Fig. 2d) and there is no correlation between the level of gene expression and H4K16ac deposition in HSCs (Fig. S2f-g), peak-calling and comparing ChIP-seq profile between samples highlighted differences in H4K16ac genomic distribution (Fig. 3a). Therefore, this dataset is still a critical part of the manuscript.**

*2. I do not see the point in plotting the tag counts around the TSS (Fig 2b) when the majority of the signal is in intronic and intergenic regions. I would suggest to plot two groups: One around the TSS (as shown) and one at all other regions centred on the centre of the peak/acetylated region. The latter will look slightly untidy as the "peaks" are very broad, but will prove the point that acetylation levels are lower in chromatin where it matters.*

**As suggested, we now also include a figure on the ChIP-seq dataset depicting all genomic regions centred on the centre of the H4K16ac peak (Fig 2c).**

*3. It would make more sense to describe the changes in nuclear shape and volume before describing the distances between chromosomes. Why the authors look at Chr 11 (Fig 2 g is seriously badly described) - would it not make more sense to already introduce Chr11 as the one that appears to carry ageing - related genes? We only learn this fact in the discussion.*

**We added more information to the description of the former Fig 2g, now Fig. 3a in the manuscript to explain the results more clearly for the readers (lines 199-203). Briefly, former Fig 2g, now Fig. 3a, shows the results of the goodness of fit test, which interrogates whether H4K16ac peaks (common in young and old+CASIN treated HSCs when compared to old HSCs, Fig. 2e) are randomly distributed within the genome. The graph displays a significant difference between a random distribution of the peaks and the observed distribution of peaks for especially chromosome 11. This data demonstrated that H4K16ac diff peaks are not randomly distributed in the genome and are selectively enriched on chromosome 11.**

*4. The Lamin studies are interesting and establish the axis CDC42 - Lamin - polarity, but they could be driven further. The manuscript entirely ignores the literature of Lamin associated domains (LADs, see this review as a good primer into the field: doi: 10.1016/j.cell.2017.04.022). LADs are associated with repressive chromatin and from this manuscript it appears as if such an association is important for HSC polarity (and quiescence?). From the DAPI stain it is already clear that the H4K16Ac staining is distinct form the more dense heterochromatic regions which should contain LADs. The relationship between the two compartments should therefore be better highlighted using additional immunofluorescence experiments.*

**As correctly stated by the referee, H4K16ac is localized at euchromatin regions and does not stain LADs. To highlight the heterochromatin compartment, we include new data showing H3K9me2 distribution within the nuclei of young, old and old CASIN treated HSCs. H3K9me2 is associated with heterochromatin regions localized at nuclear periphery and it is generally considered a marker for LADs [1]. Interestingly, consistent with a decrease in LaminA/C expression, H3K9me2 localization at nuclear periphery is clearly altered in old HSCs and is re-established at the peripheral heterochromatin compartment after treatment with CASIN,**

**which increases also LaminA/C expression. We include now these new data set in the manuscript (please see Fig 5g-h and Fig. S5a and lines 275-279).**

*5. The discussion/conclusions should be focussed on the main points as I summarize above and discuss the Lamin literature in detail and not outline why some experiments cannot be done - I agree with all that is said but that is the subject of a rebuttal letter*

**We now dicuss LaminA/C literature in more detail in the Conclusions and we have removed technical explanations/limitations of our experiments from the text (please see lines 350- 355).**

*6. The abstract is really sloppily written and does not really summarize the findings. It needs restructuring, maybe along the lines of my summary above?*

**We have re-written the abstract to better summarized the data outlined in the manuscript (please see lines 28-30 and lines 32-37).**

*Minor points:*

*1. The Figures are awfully dense. I don't know what the actual format is, but I would suggest to generate more figures and/or move some of the controls into the Supplements. I also seriously dislike it when a sentence is interrupted by a lengthy description of what is in the supplements - see page 5, line 86 - 89). Phrase properly.*

**We rephrased the description to Fig. S1a in the main text. Please see now lines 90-92. In addition, we increased the number of main figures to improve clarity and accessibility of the data for the readers.**

*2. Fig. 1 o - t: the legend of which bar describes what is tucked onto 1t, but is relevant for all panels - except s. Please place underneath o - r (horizontally)*

**The legend is now placed underneath panels o-t of Fig.1, as suggested.**

*3. The manuscript contains a multitude of grammatical errors and funny sentences, examples are:*

*Abstract "Here, we show that changes in H4K16ac upon aging and rejuvenation of HSCs are underlay to a great extent by a shift of chromosome 11 architecture.."*

*Background section: For example, it was reported an emergence 54 of bivalent domains marked by both H3K4me3 and H3K27me3 in aged HSCs compared to young [14]*

*And so on. Please ask a native speaker to go through the text*

*I hope that the author finds my comments helpful*

*Reviewer #2:*

*Grigoryan et al report that HSCs exhibit polar distribution of H4K16Ac in young but not old mice; that PTM polarity is rare; that CASIN (a Cdc42 inhibitor), but not NaB, treatment, of aged HSCs restores H4K16Ac polarity, and that this polarity is stably inherited after transplantation such that the CASIN-treated old HSCs reconstitute hematopoiesis similar to young HSCs and have H4K16Ac polarity after transplant. A link specifically to chromosome 11 is reported. The authors investigate nuclear shape and volume, observing larger nuclei with fewer invaginations upon HSC aging, which again were rescued upon treating old HSCs*

*with CASIN. These findings suggest that more general changes in nuclear structure occur during aging, so the authors explored nuclear lamins as potential mediators of the aging phenotypes. LaminA/C, but not LaminB, deletion caused changes in nuclear architecture and a pre-aging-like phenotype. These are interesting findings that build upon the group's previous work. The authors make a considerable effort to characterize several nuclear and*

*chromatin parameters in young and old HSCs and provide evidence for several selective changes with age. I agree with the authors' assertion that it is difficult (impossible?) to directly manipulate chromosome localization. In the end, though, because most changes are correlative, I have a difficult time understanding the causal relationships or how it is even possible that two sister chromosomes are co-localized to one side of the nucleus selectively in young HSCs and how this is affected via Cdc42-mediated changes to H4K16Ac.*

**We thank the referee for his/her attention in reviewing our manuscript. We are glad that he/she finds our data interesting and we are confident to have clarified and clearly substantiate our findings in the revised manuscript.**

*Specific points and suggestions:*

** P 7: The 3 patterns described do not make sense since H3K27Ac is not significantly different between HSCs and more differentiated cells (Figure 1h).*

**Indeed, only H4K5ac is significantly more enriched in HSCs compared to progenitors. Please see now lines 106-110.**

** Please be consistent with the histone modification labeling; Figure S1b uses"Me1H3K4" whereas S1c uses "H3K4Me1", etc.*

**We check throughout the manuscript to be consistent with histone modification labelling.**

** Figure 1j, k; Figure S1k: preferable to use brackets (as in Figure 1o,p,q,r, etc) to indicate which comparisons are different.*

**We now use brackets in these figures to indicate which comparisons are different (see Fig 1h, j-k and Fig. S1k).**

** P 9: "distribution of H4K16Ac is at least necessary…" needs to be rephrased. One cannot conclude, at this point, that H4K16Ac repolarization is necessary, and the sentence is awkwardly phrased, as are several others. The conclusion "..epipolarity in young HSCs and its loss upon aging is definitely not common among histone PTMs…" and yet the author's report changes in epipolarity, albeit at a smaller extent compared to H4K16Ac, in 4 out of 6*

*histone modifications assayed. It may be better to describe the H4K16Ac changes as "selective" rather than "specific".*

**We rephrased the two sentences and now we describe the changes in H4K16ac as selective. See lines 98-99 and lines 143-144.**

** If the epipolarity changes associated with aging are primarily manifested on chromosome 11, how do the authors envision that this results in global (involving many/all additional chromosomes) changes in (H4K16Ac) epipolarity, nuclear shape and volume, etc, and in a CASIN-mediated "rejuvenation" phenotype?*

**Based on full paint FISH staining, the data show changes in proximity of chromosome 11 homologues (Fig 3e-h). We did not detect changes in localization of other chromosomes (we analysed chromosomes 3, 7 and 16 beside 11; Fig 3b-d). The changes in H4K16ac epipolarity and nuclear shape and volume do not exclude in our opinion a selective involvement of alterations in chromosome 11 homolog proximity. This is now discussed in lines 329-331.**

** P9: please describe the sample preparation for ChIPseq more clearly: were all samples incubated in vitro? Was a comparison to freshly isolated cells made? Additionally, given the very large difference in overall signal intensity between young and old in Figure 2b, it is counterintuitive that only ~1300 differential peaks were found between young and aged, and that as many as ~1100 were found in aged versus aged+CASIN treated samples where the signal intensities are very similar. Please explain.*

**For all our ChIP-seq experiments, HSCs (n=3 different biological repeats each sample set, 50 000 HSCs/sample) were incubated 16 hrs in HBSS+10%FBS without growth factor at 5% CO2 and 3%O2 levels. This information is now included in the manuscript. Please see legend of Fig. 2, lines 599-600. These culturing conditions preserve HSC quiescence and function (as measured via transplantation assays) [2, 3]. As for H4K16ac, we can’t exclude alteration during this brief culturing period. Many others reported epigenetic alterations due to culturing conditions. However, we did not detect differences in H4K16ac epipolarity between freshly isolated and cultured HSCs (Supplemental data for referee, Fig. 1a).**

**Eventually, the referee is correctly observing that, given the large difference in signal intensity between young and old HSCs, is counterintuitive to only detect around 1300 differential peaks. This is due to untidiness of H4K16ac signal, particularly in old HSCs, which made the analysis challenging with respect to peak calling and identification of differential bound regions. It is known that some histone PTMs might be difficult to peak-call with the current available tools [4]. In our dataset, the overall signal intensity was higher in the ChIP-seq data set from young HSCs but there was also higher background. This affected the number of peaks called as differential across the different conditions. The peak caller tool takes into account the signal intensity at the respective peak positions as well as the signal intensity in the surrounding regions and together, these parameters affect the sensitivity of differential peak calling. The overall low and broad level of H4K16ac in old HSCs determines a bias in the diff peak analysis and we were able to conclude on a significant difference only on a rather small set of H4K16ac associated regions. To help with this aspect, we include new plots of the ChIP-seq dataset depicting all genomic regions centred on the centre of the H4K16ac peak (Fig 2c), as suggested by referee 1.**

** P 9: What does it mean that genes linked to both self-renewal, differentiation, and leukemogenesis were identified by ChIPseq? Would it not make sense if either self-renewal or differentiation were enriched, but not both? The observation of one or a couple of genes (Figure 2f) that are both differential by ChIPseq and by expression does not substantiate the conclusion that "H4K16Ac marks are linked to expression differences". Which loci are increasing in H4K16Ac binding after NaB treatment? Is this specific to CASIN treatment that when comparing CASIN to Old mice? The conclusions made from the ChIPseq and expression data in Figure 2 should be substantiated by more global, objective analyses rather than based on a few randomly selected genes. Identifying the genes that are unique to CASIN treatment when compared to NaB treatment might narrow down the mechanism of apparent regeneration after CASIN treatment.*

**We include now a more comprehensive analysis of H4K16ac ChIP-seq-associated genes based on whole transcriptome data sets. The results show no significant correlation of gene expression (both for overall expression and differentially expressed genes) and H4K16ac mark deposition in young and aged CASIN-treated HSCs compared to aged HSCs (Fig 2f and Fig. S2d-i and lines 149-150 and lines 159-179). Unfortunately, we did not perform ChIP-seq on NaB-treated old HSCs. HSCs are a very rare cell population (0.005% of total bone marrow cells in the mouse) and we pooled more than 20 mice to generate one single sample (n=3, with 50¢000 HSCs each sample). Since the transplantation assays did not show any functional improvement of NaB-treated old HSCs, we did not move forward with preparing and sequencing these sample sets.**

** Figure 2h and many others lack information on independent biological repeats, and some that do have this information have fewer than 3 independent repeats. Most, if not all, conclusions should be based on results from at least three independent experiments, and this should be clearly indicated in the legends.*

**We performed some additional repetitions of all the experiments missing at least 3 independent biological repeats and updated the data and the legends. Now all our data are based on at least 3 independent biological repeats (at least 3 mice for each sample set) and this information is included in the respective figure legends.**

** P 9: why was DNA-FISH performed on cells maintained ex vivo rather than on freshly isolated cells? Was a comparison made between cultured and fresh cells?*

**We are treating old HSCs with CASIN for 16hrs to rejuvenate HSC function alongside the epigenetic and chromatin architecture, as we show here, in agreement with previous and recent reports [2, 5]. However, we also compared DNA-FISH analysis of HSCs incubated 16hrs with freshly isolated cells and control young and old HSCs did not differ compared to the same samples cultured for 16hrs in medium without growth factors at 3%O2 levels (Supplemental data for referee, Fig. 1b).**

** P 10: The conclusion, "These data demonstrate that H4K16Ac marks are linked to expression differences of gene loci important for hematopoiesis, implying that changes in specific gene loci…" is a stretch as only 3 genes were probed for expression differences. A more in-depth analysis of expression differences and H4K16Ac differences in genes specific to HSC function would strengthen the conclusion made.*

**We include now a more comprehensive analysis of H4K16ac ChIP-seq-associated genes based on whole transcriptome data sets. The results show no significant correlation of gene expression in general (both for overall expression and differentially expressed genes) and H4K16ac mark deposition in young and aged CASIN-treated HSCs compared to aged HSCs (Fig 2f and Fig. S2d-i and lines 149-150 and lines 159-179). In addition, in the manuscript we conclude that, at least for a small number of selected genes, H4K16ac peaks can still be correlated with defined transcriptional changes. We rephrased the sentence. Please see lines 185-188.**

** Is Cdc42 inhibition known to alter gene expression and are the expression profiles revealed here similar to previous report? Cdc42 has been reported to regulate a range of HSC activities (migration, proliferation, etc): how does that fit with the epipolarity findings described here?*

**To our knowledge there are no other reports on changes of expression profiles in response to changes in Cdc42 activity. Cdc42 is not a transcription factor, but as firstly reported now in our manuscript inhibition of Cdc42 activity alters gene expression. Cdc42 has been reported to regulate HSC quiescence and bone marrow niche interaction[6], myelopoiesis and erythropoiesis[7], migration, homing[8], genomic instability and aging[2, 9]. The present findings on epipolarity open a new perspective to explain the long-term and pervasive effect linked to the alteration of Cdc42 activity.**

** Figure 3a-c: same questions as posed above for ChIPseq samples: were all samples incubated in vitro? Was a comparison to freshly isolated cells made?*

**Data depicted in former Fig. 3a-c, now Fig. 4a-c, are related to HSCs cultured 16hrs without growth factor at 3%O2. We provide now additional novel data on freshly isolated cells (Supplemental data for referee, Fig. 1c-d). We did not observe differences in nuclear volume and shape due to our culturing conditions.**

** Figure 4b: can the authors implement a quantitative measure that substantiates the statement that the LaminA/C preferentially localize near nuclear invaginations?*

**A quantification of the localization of LaminA/C at nuclear invaginations has been included in the manuscript. Please see Fig. 5d-f and lines 273-274.**

** The link between LaminA/C deficiency and aging is a bit tenuous. Have the authors investigated the aging phenotype in LaminA/C-/- mice older than 11 months (18-24 months)? Is the phenotype progressively worse? Have LaminA/C-/- HSCs from aged mice been transplanted to test functionality? If the authors have such experiments started or mice of age available, the results could potentially add significant weight to the current conclusions.*

**We agree with the referee in that the link between decrease LaminA/C expression and aging is tenuous. Along this line, in the manuscript we do not overemphasize it and we refer to *Lmna*Δ/Δ/Vav-Cre mice as showing segmental or partial premature aging-like phenotypes (Fig. 6l). Indeed, we did not expect that the *Lmna*Δ/Δ/Vav-Cre mice would have perfectly mimicked physiological aging. They are used as a tool to mechanistically address some of the epigenetic changes in old HSCs and to establish a novel Cdc42-LaminAC axis. Unfortunately, we did not aged *Lmna*Δ/Δ/Vav-Cre mice more than 11-12 months. However, we performed transplantation experiments. The data are supporting consistently a tenuous premature-aging like phenotype (Supplemental data for referees, Fig. 2) that is manifested only after secondary transplants. Partially, we explain these results because of the segmental premature-aging-like phenotype (that does not cover the whole complexity of the physiological aging phenotype) and partially we account these results to the incomplete knock-out of LaminAC in our system (see please Fig. S5d-f). We state these aspects more clearly now in the manuscript (lines 286-287).**

** The authors conclude that nuclear structure contributes to the aging phenotype, but the link to Cdc42 was not clear. Was the expression level or protein level of Cdc42 measured in the Lamin A knockout? Does CASIN treatment rescue the phenotype in the Lamin A knockout animals? Is there an additive effect if both Lamin A and Cdc42 were knocked out? Can the aging defects be rescued by restoring the expression of LaminA/C to young levels?*

**Our data reveal that Cdc42 activity (Cdc42-GTP or active levels compared to Cdc42-GDP or inactive levels) regulates the expression of LaminA/C in HSCs. Previously, it was shown that the genetic increase of Cdc42 activity via the Cdc42GAP knock-out mouse model results in a dramatic premature aging-like phenotype [9]. As well, we demonstrated previously that upon physiological aging Cdc42 activity levels are increased in the hematopoietic system and causally linked to the functional impairment of aged HSCs [2, 3]. Here, we show that LaminA/C expression is downstream of Cdc42 activity by analysing Cdc42GAP knock-out HSCs (Fig. 4h-i) and that CASIN treatment has no effect on epipolarity and nuclear volume in LaminA/C knockout HSCs (see new generated data in Fig 6d-e and Fig. S6a). To clarify the link between LaminA/C expression and Cdc42 we have rearranged some of the data (Fig. 4h-i and lines 260-262) and included the new dataset of CASIN-treated Lamin A/C knockout HSCs (Fig. 6d-e and Fig. S6a and lines 296-299).**

** Figure 5: Some clarification as to why Tubulin distribution was investigated and interpretation of the results observed in the Lamin A/C knockout are needed.*

**Tubulin distribution in former Fig. 5d was investigated as a marker for cytoskeletal polarity, as previously reported[2, 3]. Tubulin localization is polar in young and aged-CASIN treated HSCs and apolar in aged HSCs. Interestingly *Lmna*Δ/Δ/Vav-Cre HSCs show, consistent with being chronologically young, a polar distribution of tubulin in the cytosol while H4K16ac is apolar in the nucleus (similar to aged HSCs). This supports that LaminA/C acts downstream of the Cdc42-tubulin polarity, altering the epigenetic and chromatin architecture in the nucleus without affecting polarity in the cytosol. This aspect might further contribute to explain the tenuous link between LaminA/C deficiency and aging (see question above).**

**Since the dependence of LaminA/C to Cdc42 activity levels is more elegantly solved by showing the results of the CASIN treatment on *Lmna*Δ/Δ/Vav-Cre HSCs (Fig. 6d-e and Fig. S6a and lines 296-299), we decided to remove the pictures showing tubulin staining. As suggested by the referee, we are confident that this improves clarity for the readers.**

**References**

1. van Steensel B, Belmont AS: **Lamina-Associated Domains: Links with Chromosome Architecture, Heterochromatin, and Gene Repression.** *Cell* 2017, **169:**780-791.

2. Florian MC, Dorr K, Niebel A, Daria D, Schrezenmeier H, Rojewski M, Filippi MD, Hasenberg A, Gunzer M, Scharffetter-Kochanek K, et al: **Cdc42 activity regulates** **hematopoietic stem cell aging and rejuvenation.** *Cell Stem Cell* 2012, **10:**520-530.

3. Florian MC, Nattamai KJ, Dorr K, Marka G, Uberle B, Vas V, Eckl C, Andra I, Schiemann M, Oostendorp RA, et al: **A canonical to non-canonical Wnt signaling** **switch in haematopoietic stem-cell ageing.** *Nature* 2013, **503:**392-396.

4. Steinhauser S, Kurzawa N, Eils R, Herrmann C: **A comprehensive comparison of tools for differential ChIP-seq analysis.** *Brief Bioinform* 2016.

5. Leins H, Mulaw M, Eiwen K, Sakk V, Liang Y, Denkinger M, Geiger H, Schirmbeck R: **Aged murine hematopoietic stem cells drive aging-associated immune** **remodeling.** *Blood* 2018.

6. Yang L, Wang L, Geiger H, Cancelas JA, Mo J, Zheng Y: **Rho GTPase Cdc42 coordinates hematopoietic stem cell quiescence and niche interaction in the bone marrow.** *Proc Natl Acad Sci U S A* 2007, **104:**5091-5096.

7. Yang L, Wang L, Kalfa TA, Cancelas JA, Shang X, Pushkaran S, Mo J, Williams DA, Zheng Y: **Cdc42 critically regulates the balance between myelopoiesis and** **erythropoiesis.** *Blood* 2007, **110:**3853-3861.

8. Zhang X, Shang X, Guo F, Murphy K, Kirby M, Kelly P, Reeves L, Smith FO, Williams DA, Zheng Y, Pang Q: **Defective homing is associated with altered Cdc42** **activity in cells from patients with Fanconi anemia group A.** *Blood* 2008, **112:**1683-1686.

9. Wang L, Yang L, Debidda M, Witte D, Zheng Y: **Cdc42 GTPase-activating protein deficiency promotes genomic instability and premature aging-like phenotypes.** *Proc Natl Acad Sci U S A* 2007, **104:**1248-1253.

**Second round of review**

Both original reviewers were unable to re-review this manuscript, so we sought the advice of a third independent reviewer, who assessed their comments and the authors’ responses and had no further comments.
